# Tissue identity is the dominant determinant of cross-species transferability of a porcine developmental programme

**DOI:** 10.1186/s12864-026-13103-0

**Published:** 2026-07-04

**Authors:** Tianyuan Liu, Rujing Lei, Ilyas M. Khan, Peter Theobald

**Affiliations:** 1 Cardiff School of Engineering, Cardiff University, The Parade, Cardiff, CF24 3AA UK; 2 Institute for Integrative Systems Biology (I2SysBio), Spanish National Research Council (CSIC), Paterna, 46980 Spain; 3 Department of Computer Science, University of Valencia, Valencia, Spain; 4 Faculty of Medicine, Health & Life Science, Swansea University Medical School, Swansea, UK

**Keywords:** Porcine development, Transcriptomic atlas, Developmental staging, Machine learning, Cross-species conservation, Gene expression, Ordinal classification, PigGTEx

## Abstract

**Background:**

Reproducibility and cross-species translation using the domestic pig (*Sus scrofa*) are limited by the lack of a standardised molecular framework for biological maturation: the pig’s developmental tempo differs substantially from the human’s, yet no tissue-resolved transcriptomic staging system exists. Synchronising porcine and human maturation is essential to move preclinical research from descriptive to predictive.

**Results:**

We built a transcriptomic atlas of porcine development across five tissues (muscle, brain, liver, blood, lung) from 1,924 PigGTEx RNA-seq profiles. A single partial-least-squares (PLS) regressor staged each tissue at its native ordinal resolution and also drove cross-species transfer and biomarker extraction. Our central result: transfer of a pig-trained developmental score to other species is dominated by tissue identity, with phylogenetic distance a weaker secondary effect. Projected onto the seven-species Cardoso-Moreira atlas, transfer was strongest for brain and heart (*ρ = 0.89*–0.92) and weakest for the labile liver and ovary (*ρ = 0.49* and 0.42). Brain and heart stayed high even from pig to chicken ($\rho \ge 0.95$ across *∼*320 Myr), whereas liver and ovary collapsed. A variance partition confirmed the ranking (organ Type-II *F = 9.0*, $p = 2\times 10^{-5}$; phylogeny significant only at the species level, Spearman *-0.76*, *p = 0.049*, *n = 7*). The score independently confirmed lung, muscle, and adipose on the human dGTEx resource (*ρ = 0.59*–0.67) against an ortholog-scramble null and three controls. Because it is fit on pig and projected onto foreign-batch atlases, the transfer cannot arise from a pig-side artefact. A web application is available at https://pigdevstage.streamlit.app.

**Conclusions:**

The porcine developmental programme transfers to humans and more distant amniotes in a tissue-dependent manner, so the pig’s value as a developmental model is set by how conserved each organ’s developmental logic is, not by phylogenetic proximity. Validated on foreign-species atlases, the transfer sidesteps the stage–study confound that bounds within-pig staging to muscle and liver. The atlas is therefore best used organ by organ: strong for conserved organs (brain, heart) and weak for labile ones (liver).

**Supplementary Information:**

The online version contains supplementary material available at https://doi.org/10.1186/s12864-026-13103-0.

## Background

The domestic pig (*Sus scrofa*) is central to modern animal science, serving as both a major agricultural species and an increasingly important model organism [1–3]. However, a critical gap undermines reproducibility across pig research: the lack of a standardised, molecular definition of developmental stage. Current staging relies on morphological criteria or chronological age, which are poor proxies for biological maturity [4]. Littermates frequently develop asynchronously, and distinct organs mature at different rates, effectively decoupling whole-body age from tissue-specific functional competence. This imprecision confounds experimental reproducibility in nutrition, growth physiology, and disease studies, where developmental stage is a critical variable [5].

Molecular approaches offer a solution. A ‘universal’ epigenetic clock trained on 185 mammalian species [6], together with porcine-specific clocks [7], now enables broad translation of biological age between humans and other mammals. Such methylation clocks, however, primarily capture the gradual, cumulative drift of CpG methylation with age rather than the abrupt transcriptional switches that mark developmental phase transitions [8–11]. Epigenetic age can therefore align two species globally even while organ-level maturation remains asynchronous: a pig is locomotor-competent at birth, yet its immune and metabolic systems lag behind the corresponding human timeline. Transcriptomic profiling complements these clocks by providing a direct, functional readout of cellular state [12, 13]. Whereas the biological meaning of individual clock CpGs remains unclear, reflecting composites of cell division, DNA damage, metabolic activity, and cell-type composition [10], transcriptomic features are directly interpretable as active gene programmes. Transcriptomic profiling thus enables *developmental clocks* that are mechanistically transparent and tuned to organ-specific maturation. Yet a tissue-resolved developmental atlas of this kind has been missing for the pig [14, 15], limiting the standardisation of protocols across research groups.

Here, we present a multi-tissue transcriptomic atlas of porcine development, integrating five major tissues (brain, liver, muscle, lung, and blood). Unlike previous descriptive studies, we build a quantitative framework that infers developmental stage directly from gene expression with a single model. We then use that model as a portable *developmental score* to address the question that most directly tests the pig’s value as a model organism: whether the molecular programme learned in the pig transfers to other species, and whether that transfer is governed by tissue identity or by phylogenetic distance. We define stages by porcine physiological milestones (weaning, the gilt pubertal window, and reproductive maturity) and, rather than merging sparsely sampled stages into coarse binary contrasts, we drop per-tissue stages with fewer than ten samples so that every tissue is staged at its native ordinal resolution. Projecting the pig-trained score onto independent developmental atlases [15, 16], we find that the programme does transfer, and that its strength is set far more by which organ is examined than by how distant the target species is. Conserved organs such as the brain and heart transfer strongly across every species tested, whereas labile organs such as the liver transfer weaklyf on average, and unevenly across species. Because the score is fit on pig and projected onto foreign-batch atlases that share none of the pig data’s technical batches, this cross-species evidence is structurally immune to the stage–study confound that otherwise bounds within-pig staging. This work establishes a physiologically anchored molecular standard for staging in pig research, accompanied by a publicly accessible web application (https://pigdevstage.streamlit.app). The standard enables precise synchronisation of experimental cohorts and improves reproducibility across studies in swine nutrition, production, and biomedical applications.

## Results

### A comprehensive transcriptomic atlas of porcine development

**Fig. 1:**
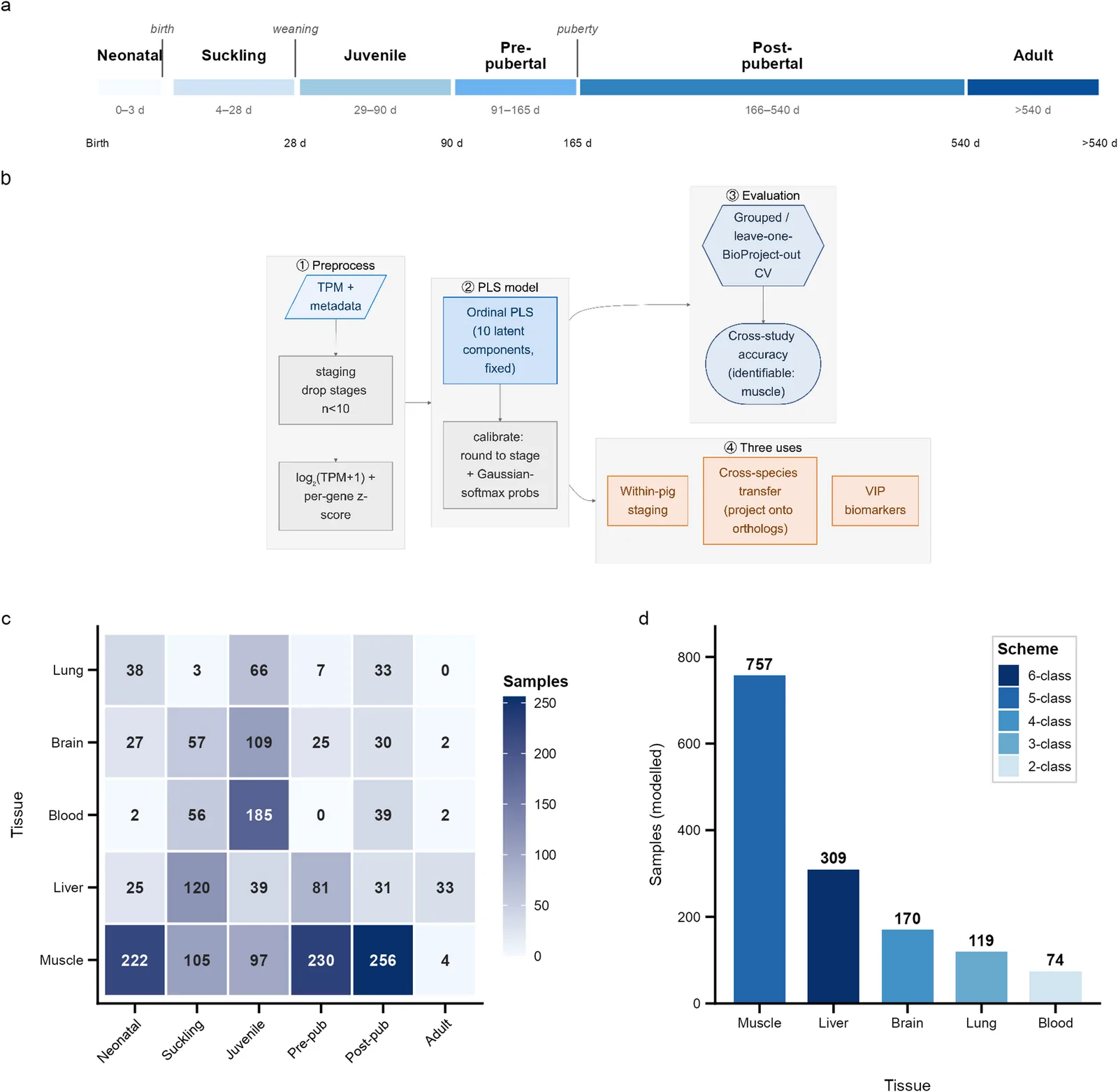
Study design and data landscape. **a**, Staging system mapping age (d) to six developmental stages anchored to physiological milestones: neonatal (0–3 d), suckling (4–28 d), juvenile (29–90 d), pre-pubertal(91–165 d), post-pubertal (166–540 d), and adult (>540 d). **b**, Machine-learning pipeline illustrating preprocessing, PLS regression model fitting (fixed ten latent components), leakage-controlled grouped cross-validation, and downstream analyses (within-pig staging, cross-species transfer, and VIP biomarkers). **c**, Heatmap showing sample counts across tissues and developmental stages (N = 1, 924 samples total). **d**, Classification tasks per tissue after dropping stages with fewer than ten samples (grey indicates dropped stages)

To define a molecular standard for porcine development, we constructed a large-scale reference atlas using RNA-seq profiles from the PigGTEx resource [17–19], a pig analogue of the human GTEx project [20] (Fig. 1c). The dataset comprises 1,924 samples spanning five major tissues (brain, liver, muscle, lung, and blood) and covering the postnatal lifespan from the day of birth to beyond 540 days of age (adulthood). Prenatal (embryonic and foetal) development is outside the scope of the present atlas: the PigGTEx resource does not include systematic, multi-tissue prenatal sampling, and addressing this developmental window would require dedicated prenatal sample collection (see Limitations). These five tissues were selected for machine-learning analysis based on postnatal sample availability and developmental stage representation.

**Fig. 2:**
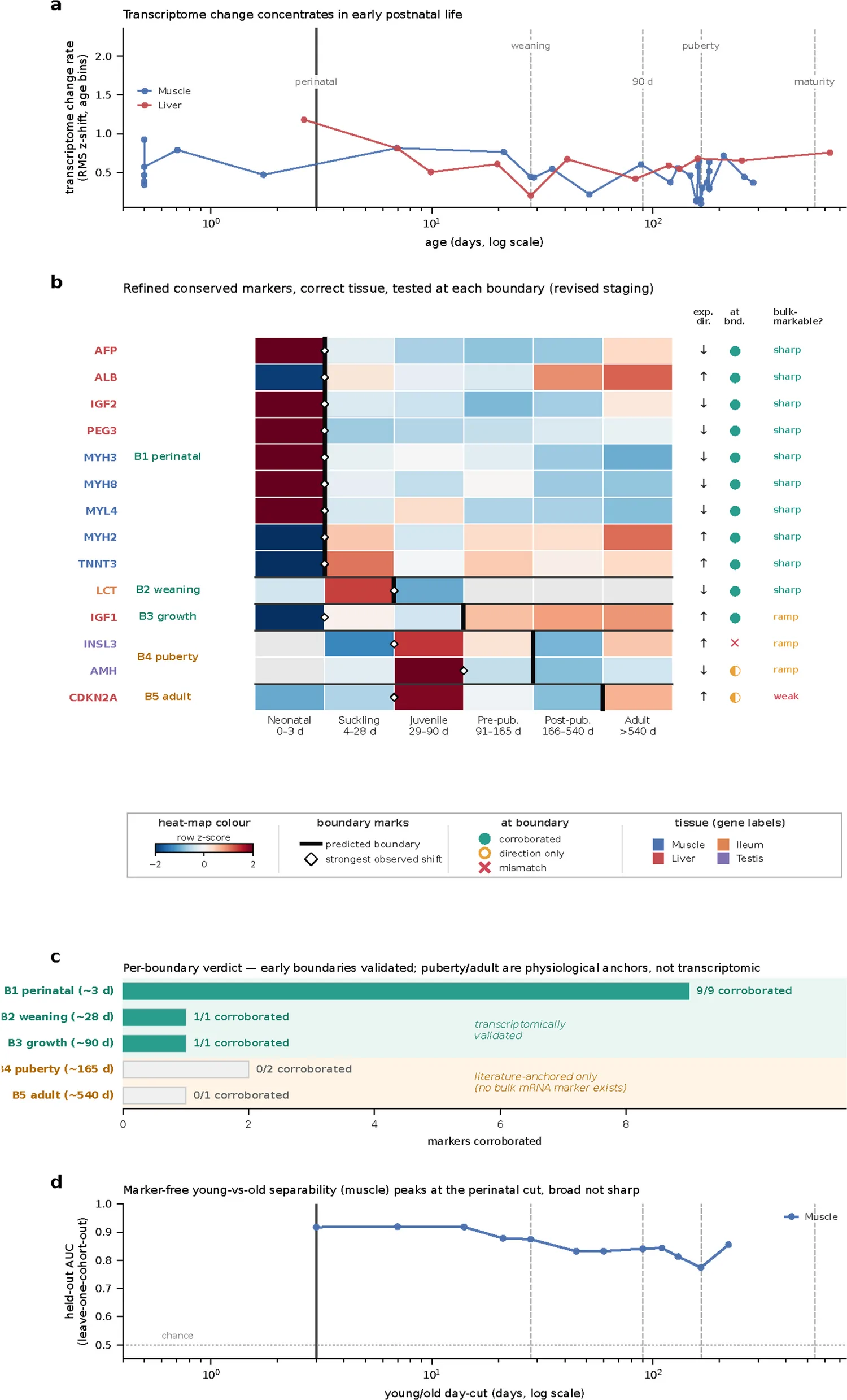
Conserved developmental markers anchor the early staging boundaries. **a** Transcriptome change rate (RMS per-gene *z*-shift across equal-count age bins) versus age (log scale), muscle and liver; dashed lines, day-cuts; solid line, perinatal boundary (*∼*3 d). **b** Row *z*-scored mean $\log _2(\text {TPM}+1)$ of the marker panel across six stages, grouped by boundary; bold lines, boundaries; diamonds, largest-fold-change transition; grey, *<3* samples. Right columns: expected direction (arrow), at-boundary significance (filled/half/cross circle *=* Tukey $p_{\textrm{adj}}<0.05$ in direction/non-significant/mismatched), pattern class; representative markers and sources in Supplementary Table S8 (Fig. 2b,c score the full 14-marker panel, of which Table S8 lists the principal anchors). **c** Per-boundary verdict (bulk mRNA): the early boundaries (perinatal, weaning, juvenile growth) are corroborated by conserved markers; the pubertal and adult boundaries are literature-defined physiological anchors with no demonstrable bulk-mRNA switch on present sampling. **d** Marker-free leave-one-cohort-out separability (muscle): held-out AUC of a genome-wide young-versus-old contrast across day-cuts (dotted line, chance 0.5), peaking at the perinatal cut (*≈*0.92 near 3 d) and declining to *≈*0.81 by *∼*130 d (broad, not sharp); cuts beyond *∼*220 d omitted (older class too sparse)

We addressed the limitations of chronological age by defining six ordered life stages anchored to porcine physiological milestones, with boundaries at 3, 28, 90, 165, and 540 days of age: neonatal (0–3 d), suckling (4–28 d), juvenile (29–90 d), pre-pubertal (91–165 d), post-pubertal (166–540 d), and adult (>540 d) (Fig. 1a). Each boundary tracks a documented transition in porcine development [1]: the perinatal surge, at which genome-wide transcriptome change is most concentrated (Fig. 2a); commercial weaning and the gastrointestinal remodelling around it [5, 21]; post-weaning growth [22]; the gilt pubertal window [23]; and reproductive maturity (Methods). Placing the first boundary at the third postnatal day isolates the steep perinatal surge that the earlier five-stage scheme had merged into suckling. Because tissues are sampled unevenly across these windows, we did not force every tissue into all six classes; instead, within each tissue we dropped any stage with fewer than ten samples and trained only on the remaining, well-supported stages (Fig. 1d). This drop-small policy preserves each tissue’s native ordinal resolution rather than collapsing sparse stages into coarse binary contrasts. We modelled developmental stage in each tissue with a single partial-least-squares (PLS) regressor, applied uniformly to within-pig staging, the cross-species transfer, and biomarker extraction (Fig. 1b; Methods).

To verify that the milestone-anchored stage boundaries correspond to genuine transcriptional transitions rather than arbitrary day cuts, we tracked canonical, evolutionarily conserved developmental markers across the six stages, each assayed in the tissue in which it is expressed, and complemented them with a marker-free leave-one-cohort-out separability scan (Fig. 2a, b). The early boundaries are corroborated by sharp, conserved, bulk-detectable switches: the perinatal boundary changes most strongly (the *MYH3/8**→**MYH2* myofibrillar switch alone spanning a >5-$\log _2$ rise), followed by weaning and juvenile growth, whereas the pubertal and adult boundaries are corroborated by no marker and remain physiological, literature-defined anchors that present-day bulk sampling cannot resolve (Fig. 2c). The marker-free criterion independently peaks at the perinatal cut (held-out muscle AUC *≈*0.92 near 3 d, declining to *≈*0.81 by *∼*130 d) but is broad rather than sharply localised (Fig. 2d). The staging is therefore a reproducible analytical scaffold consistent with the molecular data at its early boundaries, not a claim that maturation occurs abruptly at the specified day cuts.

### Tissue identity is the dominant determinant of cross-species transfer

**Fig. 3:**
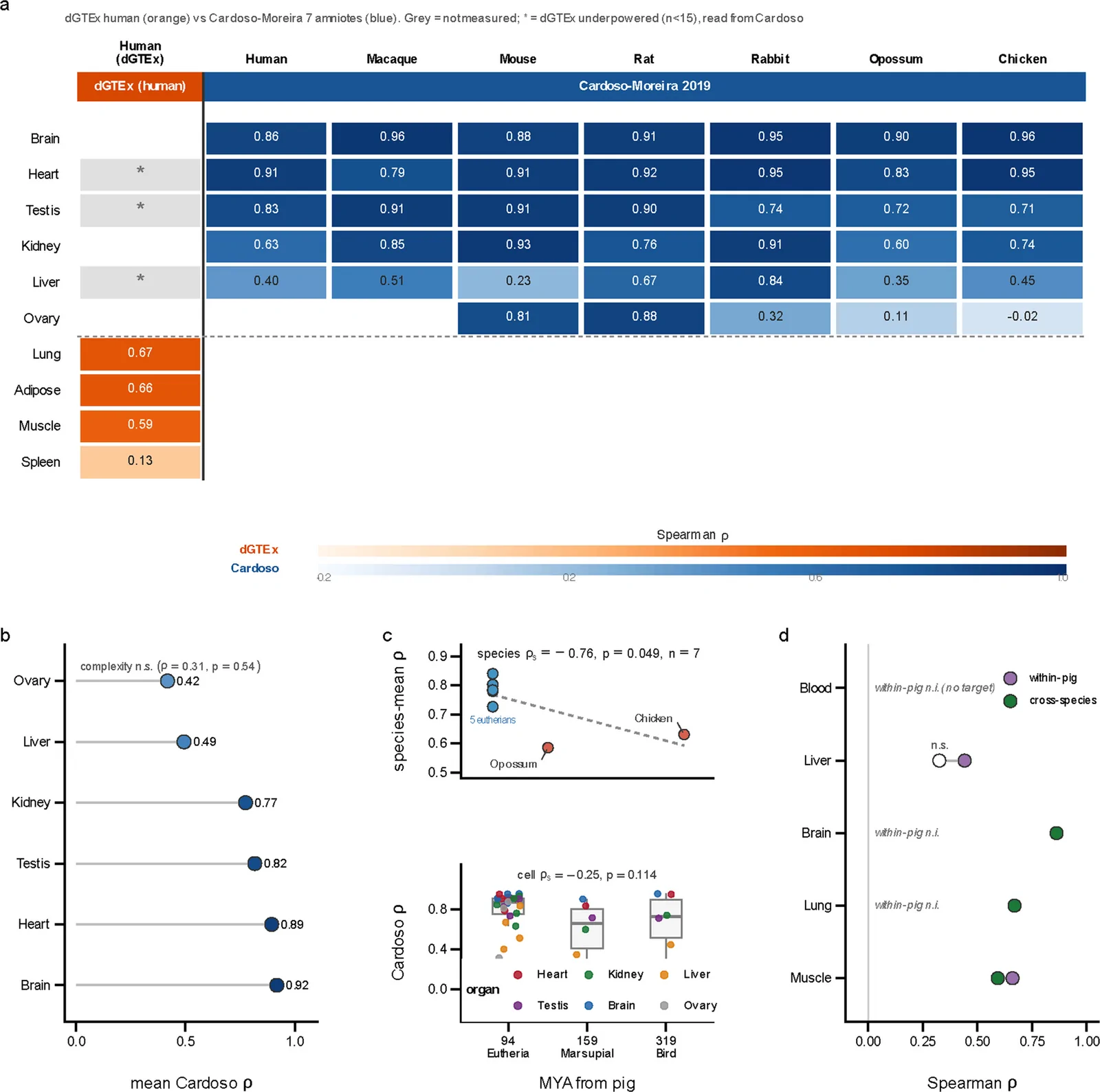
Cross-species transfer of the porcine developmental programme is determined primarily by tissue identity, with phylogenetic distance a weaker secondary effect. **a** Unified transfer matrix showing pig-to-target ordinal Spearman correlation *ρ* projecting the pig PLS score onto dGTEx (left; human) and the Cardoso-Moreira atlas (right; seven species). Rows are organs; asterisks indicate underpowered dGTEx tissues (*n < 15*). **b** Tissue effect (H2): mean Cardoso transfer *ρ* per organ, showing no correlation with transcriptome complexity (Spearman *ρ = 0.31*, *p = 0.54*, *n=6*). **c** Distance effect (H1), shown as a stacked pair on a shared divergence axis (Mya from pig; Eutheria 94, Marsupial 159, Bird 319). *Top*, the principled species-level test: each species’ mean transfer *ρ* versus divergence (the five eutherians share the 94 Mya bin; deep-divergence species labelled), with a dashed linear fit and the species-level Spearman correlation (*-0.76*, *p=0.049*, *n=7*). *Bottom*, the pseudoreplicated cell-level view: per-cell transfer *ρ* by divergence bin, coloured by organ, annotated with the cell-level correlation (*-0.25*, *p=0.11*, *n=40*). Both are shown so the borderline-significant species-level result and the non-significant cell-level result are reported together. **d** Batch-immune rescue (H3): within-pig grouped cross-validation correlation (purple) versus cross-species transfer to human (green). The within-pig grouped estimate is identifiable only for muscle and liver, whose developmental stages span multiple studies; for lung, brain and blood, the high study confounding makes the grouped estimate unidentifiable or unreliable (*n.i.*) and only the batch-immune cross-species transfer is shown (blood additionally has no cross-species target). For the cross-species (green) points, a filled circle passes the ortholog-scramble null and the open circle is the one non-significant transfer (liver). Lines connect the paired within-pig and cross-species estimates. Full transfer details are in Supplementary Table S5

The most direct test of the pig’s value as a developmental model is whether its molecular programme transfers to other species, and whether that transfer is set by tissue identity or by phylogenetic distance. We *transferred* the pig-trained PLS developmental score onto two independent atlases through symbol-based one-to-one orthologs. The first is the seven-species Cardoso-Moreira atlas [16] (human, macaque, mouse, rat, rabbit, opossum, chicken across six organs); the second is the design-matched human dGTEx cohort [15] (Fig. 3; Methods). Because the score is fit on pig and projected onto a foreign-batch atlas that shares none of PigGTEx’s technical batches, a positive transfer cannot arise from a pig-side technical artefact, and the transfer is therefore structurally immune to the within-pig stage–study confound examined below. We scored transfer as the pig-to-target ordinal Spearman *ρ*, evaluated against a 1,000*×* ortholog-scramble null.

#### Tissue dominates the transfer

Across the Cardoso-Moreira atlas, tissue-mean transfer was strongest for heart (*ρ = 0.89*) and brain (*ρ = 0.92*), intermediate for testis (*ρ = 0.82*) and kidney (*ρ = 0.77*), and weak for the labile liver (*ρ = 0.49*) and ovary (*ρ = 0.42*) (Fig. 3a,b), with the brain model defined using forebrain-aggregated subregions to match target-atlas anatomy. Brain and heart transferred uniformly across all seven species (brain *ρ = 0.86*–0.96, heart *ρ = 0.79*–0.95), and the weakest cells were not the most distant species (lowest brain cell human, *ρ = 0.86*; lowest heart cell macaque, *ρ = 0.79*), so transfer did not track phylogenetic proximity. Both survived the deepest split sampled, transferring from pig to chicken at *ρ = 0.96* (brain) and 0.95 (heart) across the *∼*320-million-year amniote divergence (ortholog-scramble *p ≤ 0.001*). Phylogenetic distance was a weaker determinant: the cell-level correlation with divergence time was modest (Spearman *ρ = -0.25*, *p = 0.11*, *n = 40*, pseudoreplicated because the five eutherian species share one divergence bin). The principled species-level test (each species’ mean transfer) was negative but only borderline-significant and rests on just seven species (Spearman *-0.76*, *p = 0.049*, *n = 7*; Fig. 3c), so we read this phylogenetic effect as tentative rather than firmly established. The organ ranking tracked neither transcriptome complexity (Spearman 0.31, *p = 0.54*, *n = 6* organs) nor target sample size (pooled Spearman 0.01, *p = 0.94*), nor pig-side training size: the labile liver has the largest pig training set of any Cardoso-Moreira organ (*n = 309*, versus *n ≤ 170* for the strongly transferring brain and heart) yet transfers weakly, so its low transfer reflects genuine developmental lability rather than limited statistical power.

Because the Cardoso-Moreira design varies *both* axes (six organs; seven species spanning divergence bins of *∼*94, 159, and 319 Mya), we partitioned the transfer variance between them. Organ identity was the dominant determinant: organ alone explained $R^2 = 0.56$ (Type-II *F = 9.0*, $p = 2 \times 10^{-5}$; df-adjusted unique $R^2 = 0.49$), far exceeding the variance a random six-level factor absorbs by chance (mean $R^2 = 0.13$, permutation *p < 0.001*). Divergence added a smaller increment (Type-II *F = 3.9*, *p = 0.056*; df-adjusted unique $R^2 = 0.04$). Transfer strength is therefore set primarily by tissue identity, with phylogenetic distance a weaker secondary effect that reaches significance only at the species level.

#### The transfer rules out a pig-side batch artefact and survives target-permutation, global-axis and organ-swap controls 

Against the ortholog-scramble null (1,000 scrambles; minimum reportable *p ≈ 0.001*), pig-to-chicken brain and heart transfer reach *ρ = 0.96* and 0.95 (*p ≤ 0.001*). After Benjamini-Hochberg FDR across the 40 Cardoso-Moreira cells, the brain (7/7), heart (6/7) and kidney (5/7) series pass at *q < 0.05* (20/40 cells overall; 24/40 at uncorrected *p < 0.05*). The labile liver and ovary fail the null in most species; testis, though its correlations are high (*ρ = 0.71*–0.91), is significant only for human and macaque; for the other species the small or unbalanced target samples (*n = 6*–22) leave the scramble null wide, so we read it as suggestive. The brain model (trained on the forebrain-aggregated subregions, *n = 170*, four stages, to match the target-species cerebrum definitions) transfers uniformly across all seven species (*ρ = 0.86*–0.96, every cell *p ≤ 0.006*), indicating a highly conserved developmental programme under anatomically matched definitions. On human dGTEx the score passes the null for the well-powered tissues, lung (*ρ = 0.67*, *p = 0.006*), muscle (*ρ = 0.59*, *p = 0.003*) and adipose (*ρ = 0.66*, *p = 0.002*), independently rescuing lung under its standard whole-organ definition. Liver transfers weakly in both substrates and blood has no cross-species substrate, so neither is validated (Fig. 3d). Because every atlas shares none of PigGTEx’s batches, this rules out a pig-side technical-batch explanation. Three further controls support a conserved developmental ordering rather than a generic gradient (Methods): the load-bearing cells stay significant under a target-label permutation null (*p < 0.001*), persist after regressing out target library size and detected-gene count (partial *ρ* 0.43–0.92), and organ-matched transfer exceeds organ-mismatched (mean *ρ* 0.63 vs. 0.25). The pre-specified substrate-routing rule (Cardoso-Moreira primary for every organ it samples; dGTEx as independent human confirmation for the tissues it powers; null-failing cells demoted to suggestive) was applied with no post-hoc re-routing (Methods). The full per-cell transfer matrix is in Supplementary Table S5 and per-cell scatter plots in Supplementary Fig. S4.

#### The transfer is not a re-detected universal proliferation axis

A natural concern is that any score declining as cell-cycle activity falls would order developmental samples in every organ, so the transfer might merely re-detect a universal proliferation-to-differentiation axis rather than a specific pig-learned programme. We tested this by benchmarking the pig PLS score against two *pig-independent* baselines computed on the identical *z*-scored shared-ortholog target matrix and ordinal level. The baselines were the target’s own first principal component ($|\rho _{\textrm{PC1}}|$, absolute because the PC sign is arbitrary) and a fixed conserved cell-cycle index (the *z*-scored mean of the Tirosh et al. S/G2M orthologs [24]; Methods). Pooled across all 47 fittable cells the three are close in mean *|ρ |* (PLS 0.71, cell-cycle 0.69, PC1 0.67; paired win-rates not significant), because in highly proliferative or labile organs a generic target-only index also aligns with the maturation axis. The decisive comparison is the tissue-identity ranking on which the central claim rests: the pig PLS score recovers the conserved*≫*labile ordering (per-organ mean *|ρ |*: brain 0.92, heart *0.89 ≫* liver 0.49, ovary 0.43). *Neither* baseline reproduces it (rank-Spearman versus the PLS ordering: PC1 *+0.03*, ranking heart last; cell-cycle *+0.09*, ranking testis last). PLS accordingly leads both within the conserved organs (brain+heart mean $\Delta |\rho | = +0.24$ versus PC1, *+0.14* versus cell-cycle) and on the well-powered human dGTEx cells (lung 0.67 versus 0.26/0.44; muscle 0.59 versus 0.28/0.53; adipose 0.66 versus 0.27/0.41), while trailing them only in the labile organs (*-0.09* versus PC1, *-0.06* versus cell-cycle). The universal-axis hypothesis predicts that the pig-independent baselines should track the PLS tissue ordering. They instead collapse it, consistent with a tissue-specific, pig-learned developmental programme rather than a generic cell-cycle axis (Supplementary Table S7).

### Cross-study staging is identifiable for muscle and liver, owing to a stage–study confound

Within the pig itself, the same PLS model’s deployability is more tightly bounded. Stages were encoded as consecutive integers and regressed on the $\log _2(\textrm{TPM}+1)$ expression matrix; a predicted stage is obtained by rounding the single real-valued PLS score to the nearest ordinal class. Because the drop-small policy retains a different number of stages in each tissue, every model operates at the resolution its sample size genuinely supports.

We distinguish two questions that compendium-staging work tends to conflate: whether developmental stage is *separable* in the transcriptome (an in-distribution question) and whether a model *deploys* to an unseen study (a cross-study question). Stratified cross-validation answers only the first, because it places samples from the same BioProject in both training and test folds. On that batch-inclusive measure stage is strongly encoded in every tissue, but each tissue retains a different number of stages *K*, so the raw balanced accuracies must be read against chance 1/*K* (liver 0.62, *K=6*; muscle 0.79, *K=5*; lung 0.96, *K=3*; blood 0.97, *K=2*; and brain 0.84, *K=4*). Every tissue sits 0.45–0.62 above its own chance level, confirming that separability is not an artefact of the coarser binary schemes (Supplementary Table S1). The deployment question requires holding out whole studies, and is identifiable for two tissues, muscle and liver; for the remaining three the sampling design precludes it.

For muscle, grouped cross-validation that holds out whole studies (StratifiedGroupKFold over the 54 muscle BioProjects, 5 folds *×* 5 seeds) recovers the developmental ordering well: the ordinal Spearman correlation between the predicted and true stage is *ρ = 0.66*, and balanced accuracy is *0.45± 0.04*, more than double the 0.20 chance rate (a ridge regressor in the same linear family agrees, 0.46). The muscle model therefore generalises across studies rather than tracking batch, and its in-distribution 0.79 falls to 0.45 once whole studies are held out (Supplementary Fig. S1a). Liver likewise orders its samples in the correct developmental direction (ordinal *ρ = 0.44*), though its balanced accuracy remains modest at *0.39± 0.06*, about *1.6×* its 0.25 chance level (Methods). Cross-study recoverability tracks the single-stage-study fraction: 47% for muscle and 84% for liver, but is very high for the remaining three tissues: 90% for brain, and 100% for blood and lung. For these latter tissues, most or all retained stages come from a single study, making cross-study staging unidentifiable or heavily confounded (Supplementary Fig. S1a; Supplementary Table S1). Their high batch-inclusive accuracies (brain 0.84, blood 0.97, lung 0.96) therefore cannot, within pig, be assigned to developmental staging rather than to separation of *studies*: with stage and study aliased, the two are indistinguishable from PigGTEx alone. Supplementary Fig. S1a instead shows the in-distribution separability, high in every tissue but not a deployment estimate. The cross-species transfer resolves this ambiguity wherever an independent substrate exists: because a model tracking only pig batch could not project onto a foreign-species atlas, the transfer of the brain and lung scores (above) is direct evidence that their within-pig accuracy rests on genuine, conserved development that PigGTEx cannot certify in isolation, whereas blood, lacking a cross-species counterpart, remains unresolved and serves as the sex-confound negative control.

The positive ordinal Spearman correlation for muscle (*ρ = 0.66*) indicates that its residual cross-study misclassifications fall predominantly between *adjacent* developmental stages, consistent with the continuous nature of postnatal maturation. For the confounded tissues the within-pig confusion structure is uninformative, as expected when stage and study cannot be separated; the evidence that their developmental programmes are real comes from the cross-species transfer above.

These differences prompted us to ask which biological programmes the muscle model, and the per-tissue feature weights more broadly, place at the centre of maturation.

### The functional landscape of development

**Fig. 4:**
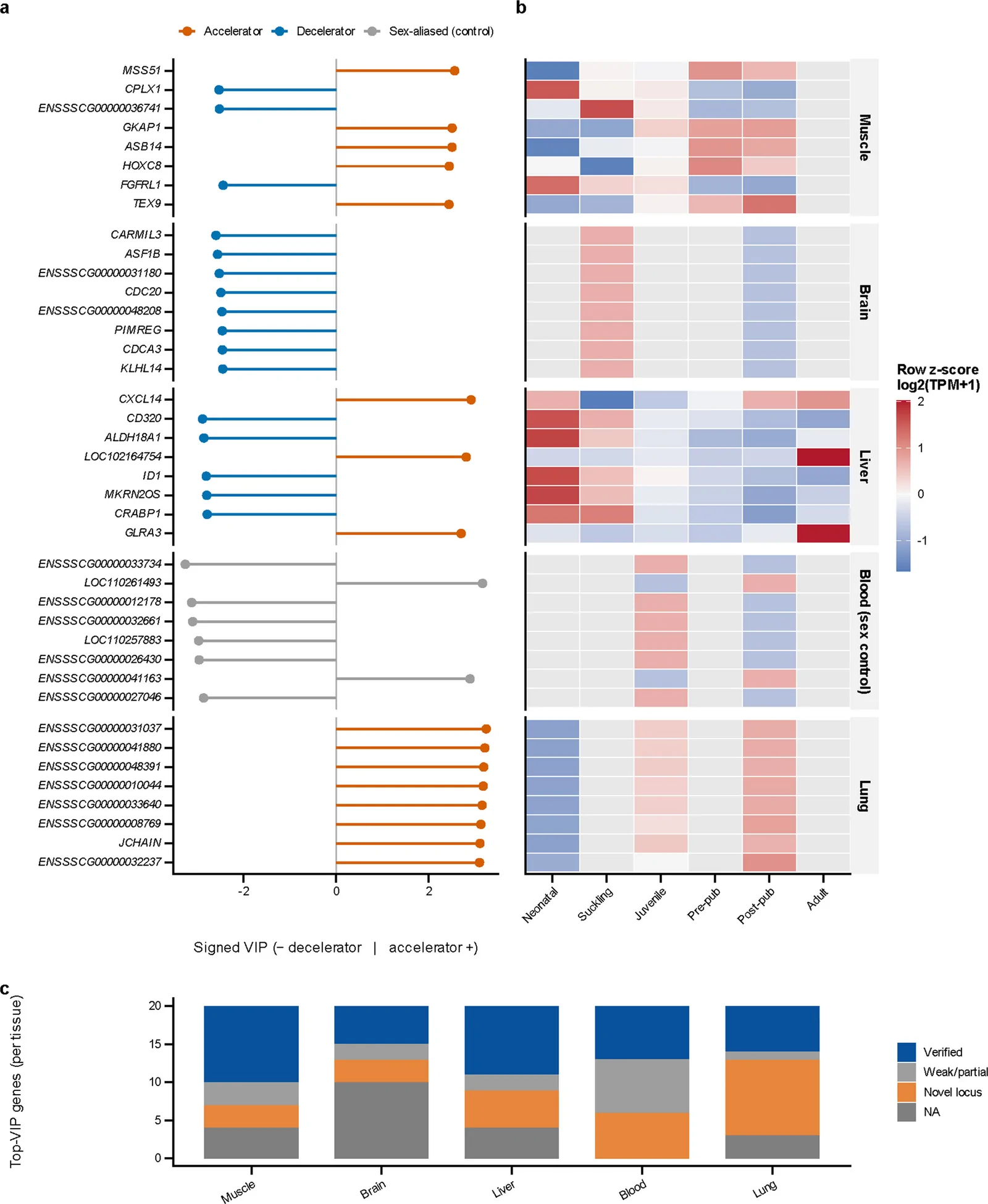
Stage-informative developmental biomarkers read from the PLS model (VIP). **a** Top 8 stage-informative genes per tissue ranked by Variable Importance in Projection (VIP), signed by trajectory direction: accelerators (orange, rising expression) and decelerators (blue, falling expression). **b** Trajectories of the same genes (shared rows with panel a) shown as row-*z*-scored mean $\log _2(\text {TPM}+1)$ expression ordered from neonatal to adult stages. **c** NCBI annotation status of the 100 top-VIP genes (20 per tissue). Blood is plotted as a sex-confound negative control dominated by Y-chromosome genes (e.g., *ZFY*, *EIF1AY*). Full results are in Supplementary Table S4

Beyond prediction, the fitted model also identifies the genes most strongly associated with maturation in each tissue. Because the staging model is a single linear projection, each tissue’s leading biomarkers, their expression dynamics across stages, and their annotation status together define a distinct regulatory programme (Fig. 4). These programmes span a contractile–metabolic maturation signature in muscle, a decline in proliferative regulators in the brain [25, 26], and an expansion of immune markers in the lung [27], with little overlap between tissues.

Feature importance rankings showed moderate run-to-run variability across bootstrap resamples (mean Jaccard similarity of the top 50 features: 0.26–0.67 across tissues; Supplementary Fig. S1b), with 24–74% of those features recurring in *≥*80% of resamples. This variability is expected for a latent-component linear model when input features are highly correlated. PLS projects the expression matrix onto a few latent components that mix co-regulated gene modules, so the specific top-ranked genes can vary across resamples while the projection, and therefore the predictions, remain stable. The selected features showed strong correlations with developmental stage (mean absolute *ρ = 0.26*–0.73; Supplementary Fig. S1c). Cross-tissue feature overlap was very low (0.7% Jaccard similarity), indicating that each tissue uses largely distinct molecular programmes for developmental progression.

Because the uniform model is a PLS regressor, each tissue’s biomarkers are read *directly from the fitted projection* rather than from a separate differential-expression test: genes are ranked by Variable Importance in Projection (VIP), and the sign of each gene’s coefficient onto the ordinal stage index labels it an *accelerator* (rising toward later stages) or *decelerator* (falling) (Supplementary Table S4; trajectories in Fig. 4b). We emphasise the leakage-robust **muscle** model first, since it is the only tissue whose accuracy survives grouped cross-validation; the other tissues’ VIP sets are reported with explicit caveats because stage is largely confounded with study.

In muscle, the top-VIP biomarkers form a coherent maturation programme. Accelerators rising through the pre-/post-pubertal window include the muscle-specific mitochondrial regulator *MSS51/ZMYND17* (VIP 2.56, the highest-ranked muscle feature; a myostatin/TGF-*β* target) [28], the ubiquitin-ligase adaptor *ASB14* [29], the homeobox transcription factor *HOXC8* [30], and the IGF-1-coupled E3 ubiquitin ligase *NEDD4* [31]. Conversely, decelerators highest in the neonatal/suckling window include complexin-1 (*CPLX1*) [32], the kinase-deficient FGF receptor *FGFRL1* (a negative regulator of proliferation) [33, 34], and the imprinted regulator *DLK1* [35]. This accelerator/decelerator split (early proliferative tone giving way to a mature contractile–metabolic phenotype) is the biologically interpretable, leakage-robust biomarker signal of the atlas. A high-ranking unannotated pig locus (*ENSSSCG00000036741*) is reported as a novel candidate rather than interpreted (Supplementary Table S4).

For the other four tissues the VIP genes still name coherent maturation programmes (full lists, directions, and per-stage expression in Supplementary Table S4); we report only online-verified, developmentally plausible roles. In brain, the verified hits are canonical cell-cycle/proliferation regulators (*CDC20*, *ASF1B*, *PIMREG*, *CDCA3*, *UBE2C*, and the securin *PTTG1*) [36–41] whose decline from early stages (neonatal/suckling) to post-pubertal matches the documented switch from neural proliferation to maturation, a signature corroborated by the strong cross-species brain transfer (above). In lung, the verified top-VIP genes are immune-maturation markers (the macrophage marker *CD68* [27]; the T-cell genes *CD3E*, *CD3D*, *CD8A*, *CD6* [42–45]; and the joining chain *JCHAIN* [46]). This programme of postnatal adaptive-immune expansion is not a pig-side batch artefact, because it is independently confirmed by transfer to human (pig-to-human *ρ = 0.67*, scramble *p = 0.006*; above). In liver, the verified hits are individually plausible developmental genes (*CRABP1* [47], *ID1* [48], *ALDH18A1* [49], *CXCL14* [50]). However, liver is the weakest of the five for biomarker interpretation: it transfers weakly across species, and two neuronal genes (*GLRA3*, *RIMS1*) [51, 52] appear as likely contamination, so we read the liver biomarkers cautiously. Blood is reported only as a sex-confound negative control: its top-VIP set is dominated by Y-chromosome genes (*ZFY*, *EIF1AY*) [53, 54], so part of its “signal” separates animal sex, and with 100% single-stage-study confounding and no cross-species substrate we do not interpret it as developmental biology. Across all five tissues a substantial fraction of the top-VIP entries are unannotated pig loci, reported as uncharacterised candidates rather than assigned invented functions.

## Discussion

We have established a multi-tissue transcriptomic atlas for porcine development and used it to test whether a pig-trained developmental score transfers to other species. It does, in a manner dominated by tissue identity, with phylogenetic distance a weaker secondary effect. Two further results frame this spine. First, a single PLS regression model infers developmental stage from gene expression. Evaluated honestly under grouped cross-validation that holds out whole studies, it generalises across studies for muscle (ordinal *ρ = 0.66*; balanced accuracy *0.45± 0.04*, more than double its 0.20 chance) and orders liver developmentally (*ρ = 0.44*; balanced accuracy 0.39; chance 0.25). In the remaining three tissues developmental stage is confounded with the contributing study by the resource’s design. Second, the cross-species transfer is generated on foreign-batch atlases and is therefore structurally immune to that confound. It remains the load-bearing evidence that the developmental signal is biological. This shift from qualitative observation to a transferable, quantitative score addresses a long-standing challenge in pig research: standardising developmental staging across experimental cohorts and research groups. To facilitate community adoption, we provide an open-access interactive web application (https://pigdevstage.streamlit.app) for uploading gene expression profiles and obtaining developmental stage predictions.

Detailed input-format requirements and preprocessing recommendations (gene-identifier handling, microarray and out-of-distribution data, and recommended gene-coverage thresholds) are provided in the application’s in-context help text. Importantly, the leakage-controlled evaluation above shows that the staging predictor is cross-study validated for muscle (robustly) and liver (more modestly); for brain, lung, and blood the model is calibrated to the cohort composition of PigGTEx and has not been shown to generalise to independent studies. Predictions for these three tissues should therefore be treated as exploratory and re-calibrated on a small in-house labelled cohort before use, and liver predictions warrant similar caution given its weaker cross-study estimate. The application surfaces this cross-study-validation status in the prediction panel.

The practical implications are direct: classifying animals by biological maturity rather than chronological age reduces confounding variability in studies of nutrition, growth physiology, and disease susceptibility. Synchronising cohorts by molecular stage should also improve statistical power and reproducibility. The tissue-specific models also let researchers choose a staging approach suited to their target organ system.

As a prospective application, the muscle score, the one tissue cross-study validated and transferable to human, could help synchronise cohorts in porcine models of age-dependent muscular disorders such as Duchenne Muscular Dystrophy (DMD) [55, 56]. We do not test disease tissue or compare species here and make no claim about porcine versus rodent fidelity; the hypothesis is simply that defining developmental stage molecularly rather than by chronological age could reduce variability in disease progression and treatment response, to be evaluated directly in a dystrophic cohort.

The profound tissue-specificity of developmental markers (0.7% cross-tissue Jaccard overlap) reflects genuine biology: each tissue uses largely distinct molecular programmes. The moderate within-tissue variability of the VIP rankings (Jaccard 0.26–0.67) is best read as gene redundancy, as noted above. Because a latent-component model spreads each co-regulated module’s contribution across the projection, the highest-VIP genes can differ across resamples while the developmental score stays stable. This parallels penalised and latent-variable regression in epigenetic-clock construction, where multivariate optimisation routinely selects age-uncorrelated CpGs from co-regulated modules without compromising prediction [10].

This tissue-dominated transfer is illustrated most clearly by the conserved cardiac and brain programmes transferring from pig to chicken across roughly 320 million years of divergence ($\rho \ge 0.95$). In that same atlas the labile liver and ovary collapsed (*ρ = 0.45* and *-0.02*) and transferred poorly even among close mammalian relatives. We read this as evidence that the pig’s value as a developmental surrogate is set by how strongly a given organ’s developmental logic is conserved, consistent with reports that cardiac and neural trajectories are comparatively constrained across amniotes whereas hepatic and gonadal trajectories diverge [16], and with work on species-specific developmental tempo explaining why conserved programmes unfold at different rates [57]. Because this transfer is generated on foreign-batch atlases, it supplies the developmental validation the within-pig evaluation cannot. It confirms muscle and rescues lung under its standard whole-organ definition (pig-to-human *ρ = 0.67*). For brain and lung, the cross-species transfer validates the developmental signal, while liver and blood remain unvalidated.

More broadly, the framework is species-agnostic: the only required inputs are bulk RNA-seq profiles with age metadata across developmental stages. Pan-mammalian epigenetic clocks have shown that biological-age prediction generalises across species [6–8]; our method shares that principle but applies ordinal classification to transcriptomic data, and the growing availability of multi-species developmental atlases [15, 16] provides the foundation for extending it to additional livestock and model species.

Training on chronological bins to capture biological age is a deliberate trade-off: in the absence of a gold standard for physiological maturity, chronological age is a noisy proxy, and consistent learning under label noise is achievable with sufficient data [58]. Ordinal classification is also more robust to boundary noise than continuous age regression. Misclassification between adjacent stages reflects genuine transitional biology rather than the regression-to-the-mean artefacts that confound continuous epigenetic clocks [10]. That the pig-trained score transfers to independent human and multi-species atlases despite vastly different chronological lifespans (Fig. 3) supports the interpretation that it captures conserved biological programmes rather than chronological associations alone.

The boundary check (Fig. 2) is a consistency test, not an independent validation of where the cuts fall: the conserved markers were chosen *because* they switch around the targeted transitions, so their concordance confirms only that bulk RNA-seq registers the expected switches, each in the tissue in which it is expressed. The hepatic-maturation [59, 60], imprinted-growth [61], embryonic-to-adult myofibrillar [62], weaning (*LCT*) [63] and juvenile-growth (*IGF1*) [64] markers all move in the predicted direction at the early boundaries (Fig. 2b,c), and the marker-free leave-one-cohort-out criterion peaks at the perinatal cut yet is broad rather than sharp (Fig. 2d). The pubertal and adult boundaries, by contrast, are corroborated by no marker and remain *physiological, literature-defined* anchors for which a transcriptomic switch-point is, on present data, neither demonstrated nor refutable, since PigGTEx barely samples the adult window. We therefore read the early boundaries as molecularly grounded and the later ones as operational, reproducible cuts rather than claimed sites of abrupt maturation.

Several limitations must be acknowledged. First, the present atlas is restricted to *postnatal* development (day 0 to >540 d); PigGTEx does not provide systematic multi-tissue prenatal RNA-seq sampling across these five tissues, and we therefore make no claim about prenatal trajectories. Extending the framework to embryonic and foetal stages will require dedicated prospective sampling across pregnancy, for which integration with single-cell atlases of porcine organogenesis is the natural complement.

Second, PigGTEx aggregates RNA-seq from multiple platforms and protocols without explicit batch correction, which we assessed in two ways. Within the two largest single-project muscle cohorts, gene-level fold-changes correlated strongly with full-dataset estimates across three developmental contrasts (pooled Pearson *r = 0.76*–0.80, genome-wide; Supplementary Table S2), confirming the signatures reflect biology even at the earliest transitions. An embedding-level decomposition of PCA and UMAP variance against Stage and BioProject (Supplementary Fig. S2; Supplementary Table S3) showed developmental stage explaining a substantial share of PC1 and producing visible neonatal-to-adult gradients in four of five tissues, liver the most batch-dominated (its developmental component recovered only at PC5). BioProject explains the larger absolute variance share in every tissue, because most projects sample a single narrow age window, so Stage and BioProject are confounded by design. This is why a stratified split inflates apparent accuracy and only the grouped split measures genuine cross-study prediction. Future work should prioritise datasets whose individual studies each span multiple developmental stages.

Third, the cross-species substrates differ in power, and we foreground the well-powered, scramble-validated cells. The seven-species Cardoso-Moreira panel carries the tissue*≫*phylogeny comparison because it varies both axes, but its divergence axis is coarse (one marsupial and one bird at the deep end) and organ-unbalanced. We therefore read the phylogenetic determinant from the species-level test and the crossed mixed model rather than the pseudoreplicated 40-cell correlation. The dGTEx panel confirms only the tissues it powers (lung, muscle, adipose); its heart (*n=7*) and testis (*n=10*) cells are underpowered and reported only in Supplementary Table S5. Because transferability is tissue-dependent, liver and ovary, which transfer weakly and fail the null in most species, warrant species-specific validation before the pig is used as their developmental surrogate.

Fourth, the within-pig staging is honestly bounded: a cross-study deployment estimate is obtainable for muscle and liver (ordinal *ρ = 0.66* and 0.44; balanced accuracy 0.45 and 0.39, against chance 0.20 and 0.25). For the remaining three tissues developmental stage is so confounded with the contributing study (single-stage-study fraction 90–*100%*) that a cross-study test is not feasible, and those models are calibrated to the PigGTEx cohort rather than shown to generalise. This bound is set by the data, not the estimator: linear comparators give similar within-pig muscle accuracy, while nonlinear estimators reach a higher within-pig ceiling that does not transfer (Supplementary Table S6; Supplementary Fig. S5). Here too the batch-independent cross-species transfer provides the developmental validation that the within-pig evaluation cannot. Finally, the staging boundaries are operational: an alternative milestone-anchored scheme yielded the same qualitative cross-species conclusions, so the result does not hinge on the exact day cuts.

Fifth, within each stage bin the cohort is not balanced for breed, sex, or BioProject, and in PigGTEx the BioProject is the strongest correlate of all three. As a complement to the grouped cross-validation, a within-stage subgroup audit (Supplementary Fig. S3) recomputed balanced accuracy within every Breed, Sex, and BioProject level satisfying *n ≥ 10*, tested against a within-stage label permutation null (1,000 shuffles). Subgroup composition matters: per-breed muscle balanced accuracy ranges from 0.56 (Yorkshire) to 0.93 (Duroc), with the deployment-relevant Large white cohort at 0.59, and under the six-stage scheme the permutation test rejects the chance null only for liver/Breed (*p<0.001*). Muscle/Sex (*p=0.35*), muscle/BioProject (*p=0.08*), muscle/Breed, brain/Sex, liver/Sex, and lung/Sex are all consistent with chance. Deployment to cohorts dominated by under-represented breeds (e.g. exclusively Large white) therefore warrants re-calibration, as flagged in the web-application documentation. Sixth, bulk RNA-seq averages signals across cell types; future single-cell iterations [65] will resolve which cell populations drive these developmental clocks.

## Conclusions

This work provides a transparent, reproducible, physiologically anchored molecular standard for developmental staging in pig research, and shows that the programme it captures transfers to other species in a tissue-dependent manner. The atlas is an immediately applicable tool for standardising experimental cohorts in swine nutrition, production, and biomedical studies. The transfer analysis adds a clear usage rule: the pig is a strong developmental surrogate for conserved organs such as the brain and heart, and a weak one for labile organs such as the liver and ovary; transferability should be judged organ by organ rather than by phylogenetic proximity. As the pig continues to serve agricultural and biomedical research alike, this resource helps define developmental age with the precision that reproducible research requires.

## Methods

### Data acquisition and processing

Bulk RNA-seq transcript-per-million (TPM) matrices and metadata were obtained from the PigGTEx resource [17, 18]. Ages recorded in days were harmonised to six ordered developmental stages with boundaries at 3, 28, 90, 165, and 540 d: neonatal (0–3 d), suckling (4–28 d), juvenile (29–90 d), pre-pubertal (91–165 d), post-pubertal (166–540 d), and adult (>540 d). These boundaries were chosen to align with established physiological milestones in porcine development [1]: the perinatal transition of the first postnatal days, at which genome-wide transcriptome change is most concentrated (Fig. 2a); the upper bound of commercial weaning (*∼*19–28 d) [5]; completion of post-weaning gut adaptation and early juvenile growth (*∼*90 d), coinciding with the onset of prepubertal ovarian follicular activation in gilts (postnatal day *∼*75–95) [22, 66]; the gilt pubertal window (*∼*75–165 d depending on genetics and management) [23]; and full reproductive maturity (>540 d). The first boundary splits the previous single infancy bin (0–28 d), isolating the perinatal transcriptional surge that the earlier five-stage scheme had merged into the suckling window.

#### Per-tissue drop-small policy

Because PigGTEx samples tissues unevenly across these windows, we did not force every tissue into all six stages. Within each tissue, any stage represented by fewer than ten samples was dropped (not merged into an adjacent stage) before model training, so that each classifier operates on a contiguous, well-supported subset of the ordinal scale at its native resolution. Under this policy liver retained all six stages, muscle five (neonatal, suckling, juvenile, pre-pubertal, post-pubertal; adult dropped, *n=4*), lung three (neonatal, juvenile, post-pubertal), blood two (juvenile, post-pubertal), and brain four (neonatal, suckling, juvenile, post-pubertal; pre-pubertal and adult dropped); per-stage sample sizes are shown in Fig. 1c, with per-tissue totals in Supplementary Table S1.

Preprocessing consisted of a $\log _2(x+1)$ transformation applied to TPM values, placing all genes on a common expression scale. We did not apply variance filtering. The PLS model standardises each gene to zero mean and unit variance internally; to avoid leakage this *z*-scoring is fit on the training fold only and applied to held-out samples, and the number of latent components (rather than a separate feature pre-selection step) controls model capacity across the full gene set.

### Machine learning framework

Developmental stage was modelled with a single Partial Least Squares (PLS) regression [67], used uniformly for within-pig staging, the cross-species transfer below, and biomarker extraction, so that all three analyses rest on one transparent linear projection. We treated the ordered stages as a numeric target and rounded the model output to the nearest class, a regression approach to ordinal classification rather than a dedicated ordinal estimator. Each developmental stage was integer-coded ($y \in \{0,\dots ,K-1\}$) and regressed on the $\log _2(\textrm{TPM}+1)$ expression matrix across all available genes (31,908 per tissue), with genes *z*-scored inside the model (fit on training folds only). The number of latent components was fixed at ten, which controls model capacity across the full gene set. A predicted stage is obtained by rounding the single real-valued PLS score $\hat{y}$ to the nearest integer class, which is non-decreasing in the score by construction, so predictions are ordinally consistent without a Frank & Hall cumulative decomposition, without $K{-}1$ binary sub-models to recombine, and without a post-hoc monotonicity projection. For reporting calibrated probabilities only, the score may optionally be mapped to a class distribution by a Gaussian-distance softmax,

1 $$\begin{aligned} \Pr (y = c \mid x) & \;\propto \; \textrm{exp}\left( -\frac{(\hat{y}-c)^2}{2\sigma ^2}\right) , \\& \qquad c \in \{0,\dots ,K-1\}, \end{aligned}$$

with *σ = 0.8*; this layer affects only the displayed probabilities and not the predicted class, balanced accuracy, F$_1$, or ordinal *ρ*, all of which derive from the rounded score. Per-tissue feature importance is the Variable Importance in Projection (VIP), computed per latent component from the model weights and normalised so that the mean squared VIP equals one (VIP *>1* flags an above-average contribution). The sign of each gene’s PLS regression coefficient onto the stage index distinguishes accelerators (+) from decelerators (−).

This single PLS model is deliberately simpler than a gradient-boosted Frank & Hall reduction [68] over LightGBM [69], which would decompose the *K*-class problem into $K{-}1$ cumulative binary subtasks. A benchmark of eighteen estimators under the identical leakage-controlled protocol (Supplementary Table S6) shows the highest within-pig muscle accuracy (the primary cross-study-identifiable tissue) is reached not by PLS but by that gradient-boosted tree (balanced accuracy 0.537 versus PLS 0.427; chance *1/K = 0.20*). Both PLS figures are the same fixed 10-component default model: the standalone benchmark harness (*n = 757*, 54 BioProjects) reports 0.427 and the main pipeline (n = 757, 54 BioProjects) 0.45 ± 0.04, agreeing within seed-level scatter. The n = 761/56-BioProject counts in Supplementary Table S3 are the pre-drop embedding superset, which includes sub-threshold dropped stages and is not the basis of these accuracies. That advantage does not transfer: a tree’s pig-specific split thresholds and feature interactions have no counterpart in a target species’ ortholog space, so every tree- and boosting-based estimator collapses on the foreign-species atlases (failing the scramble null for all organs except muscle). By contrast, the linear-projection family carries the developmental signal across species (Supplementary Fig. S5; Supplementary Table S6). We therefore adopt PLS regression as the single uniform model: among the transfer-capable linear models it is the highest-scoring one that also yields VIP biomarkers, it is order-aware, and it runs at a fraction of the boosted trees’ memory and compute; the tree’s higher within-pig accuracy is reported as a non-transferable ceiling. Within-pig accuracy was thus not the selection criterion, and in any case the honest scores (0.427 for PLS, 0.515 for PCA+ridge, 0.537 for the tree) all sit far below the leakage-inflated stratified estimates the same models reach (0.75–0.92, collapsing to 0.36–0.54 once whole studies are held out; Supplementary Table S6), so the small gaps among them carry little weight. Order-awareness matters because balanced accuracy is order-blind, whereas a developmental stager should keep its errors near the true stage. That the deployed PLS does so is confirmed by its ordinal Spearman on muscle (*ρ = 0.66* under grouped cross-validation). PLS-regression is preferred over PLS-DA because only the continuous PLS score is projected across species. Because transfer-capability is an explicit selection criterion, the cross-species conservation we report is confirmatory *given* a linear projection, and is a property of the model *class* rather than any single fit.

Models were evaluated under leakage-controlled *grouped* cross-validation as the primary protocol. The outer loop used StratifiedGroupKFold grouping on BioProject (*K = 5*, automatically reduced for tissues with small class sizes), so that no BioProject contributes samples to both the training and test folds. Because a grouped split removes an entire study from training, a developmental stage contributed by only a single BioProject cannot be evaluated cross-study; the grouped balanced accuracy was therefore computed over the stages present in at least two BioProjects. For muscle this is all five retained stages; for liver it excludes the neonatal and adult stages (each drawn from a single dominant BioProject), leaving four (chance 0.25); for lung, blood, and brain, the high study confounding (90–100% single-stage representation) prevents reliable cross-study evaluation. For each seed, balanced accuracy and Macro-F1 were averaged across the held-out folds, while the ordinal Spearman *ρ* and per-class recall were computed on that seed’s pooled held-out predictions; all are reported as mean ± SD across the 5 seedsThe number of latent components was fixed at ten (no per-fold tuning with Optuna [70]; preprocessing and metrics were otherwise identical between the grouped and stratified protocols.Stratified *K*-fold cross-validation is reported only as the in-distribution, batch-inclusive contrast, and the stage–study confound was quantified per tissue as the fraction of retained samples originating from single-stage BioProjects. A final model was retrained on all available samples for the transfer and VIP analyses. Primary performance metrics included Balanced Accuracy (BA; Supplementary Fig. S1a; Supplementary Table S1) and Macro-F1 score:

2 $$\begin{aligned} \textrm{BA} = \frac{1}{K} \sum \limits _{c=0}^{K-1} \frac{\textrm{TP}_c}{\textrm{TP}_c + \textrm{FN}_c} \end{aligned}$$

Confidence intervals (95%) were calculated using bootstrap resampling (1,000 iterations) on the pooled cross-validated predictions.

### Cross-species developmental-score transfer

To test whether the porcine developmental programme transfers to other species, we transferred the pig-trained PLS developmental score onto independent species atlases rather than comparing static fold changes. For each organ, a pig PLS model (the same model family used for within-pig staging) was fit on that organ’s PigGTEx developmental samples under the identical six-stage drop-small policy (pig training *n* ranging from 32 for kidney to 757 for muscle, with brain defined as the forebrain-aggregated construct, *n = 170*, four stages; per-organ pig *n* and retained stages are listed in Supplementary Table S5). Its real-valued developmental score was then projected, unchanged, onto the matched genes of the corresponding organ in an independent target-species developmental matrix, yielding one projected score per target sample. Pig and target features were *z*-scored by *independent* scalers fit on disjoint data, and no gene was selected using target labels. Transfer was quantified as the Spearman correlation between the projected pig score and the target species’ own ordinal developmental level (pig-to-target ordinal Spearman *ρ*); a positive *ρ* means the pig-trained score orders the target samples in the correct developmental direction. Robustness to target normalisation was confirmed by repeating each transfer with per-sample *z*-scoring and with a split-half target scaler.

#### Ortholog mapping (symbol-based one-to-one)

Pig (*Sus scrofa*) and target-species Ensembl identifiers were resolved to gene symbols via mygene.info, retaining only symbols appearing *exactly once* in each species’ annotation so that every transferred feature is a one-to-one ortholog; transfer was computed on the per-species intersection of these shared-unique symbols (human dGTEx $n \approx 15{,}200$ shared symbols; human Cardoso-Moreira $n \approx 15{,}200$; down to chicken $n \approx 5{,}951$).

#### Ortholog-scramble null

To establish significance independent of the small per-tissue target sample sizes, each observed transfer *ρ* was compared to a null generated by permuting the ortholog mapping (shuffling which target gene column receives which pig weight) 1,000 times and recomputing the transfer score, with an empirical *p*-value the fraction of scrambled mappings reaching the observed *ρ* (per-cell results in Supplementary Table S5). The underpowered human dGTEx heart (*n=7*) and testis (*n=10*) cells were flagged: dGTEx heart fails the scramble null (*p=0.584*) and rides a global library-size*∼*age confound. Heart conservation is therefore reported from the well-powered Cardoso-Moreira values, and testis is treated as suggestive; the dGTEx heart and testis *ρ* appear only in Supplementary Table S5 with their *n*. Because the scramble null holds the target labels fixed and tests only the gene alignment, we ran a complementary *target-label permutation null* for the load-bearing cells, shuffling the target ordinal developmental labels 1,000 times (gene alignment held fixed); all five well-powered cells remained significant (dGTEx lung, muscle and adipose, Cardoso-Moreira heart, and brain, each *p<0.001*). To exclude a generic bulk-composition gradient we additionally (a) regressed target library size and detected-gene count out of both the projected score and the target ordinal level (partial-Spearman residual *ρ* 0.43–0.92, close to the raw *ρ*), and (b) ran an organ-swap negative control, projecting each pig-organ score onto every target organ (organ-matched transfer exceeded organ-mismatched, mean *ρ* 0.63 vs. 0.25). Some pig organs, such as heart and kidney, carry a shared cross-organ maturation component, so the swap supports an organ-specific signal without fully isolating it.

#### Pig-independent baselines and a random-set floor

To test whether the transfer merely re-detects a universal proliferation axis, each cell’s pig-trained PLS score was compared against two baselines computed on the *identical* *z*-scored shared-ortholog target matrix and ordinal level, using no pig information: (i) a target-only first principal component (PCA fit unsupervised on the target matrix; $|\rho _{\textrm{PC1}}|$ used because the PC sign is arbitrary), and (ii) a fixed conserved cell-cycle index (the equal-weighted mean of the *z*-scored one-to-one orthologs of the Tirosh et al. S/G2M signature [24], 96 symbols; $n_{\textrm{CC}}$ per cell ranging 51 in chicken to 90 in mouse). As a null floor, the same scoring was repeated for 1,000 random gene sets of matched size per cell. Sharing the target matrix and level vector across methods isolates what the pig-learned weighting adds over a pig-independent index (Supplementary Table S7).

#### Target developmental atlases

We applied the transfer test to two substrates. (i) The seven-species developmental atlas of Cardoso-Moreira et al. [16] (E-MTAB-6814; human, rhesus macaque, mouse, rat, rabbit, opossum, chicken across cerebrum, cerebellum, heart, kidney, liver, ovary, and testis), which varies both organ identity and phylogenetic distance and so carries the comparative analysis of tissue and phylogeny as determinants of transfer. (ii) The human developmental GTEx (dGTEx) resource [15], design-matched to our analysis (paediatric-to-adult, multi-tissue), used as an independent confirmation for the tissues it samples with adequate *n* (lung, muscle, adipose). The substrate-routing rule was pre-specified before results were inspected: the Cardoso-Moreira panel is the primary substrate for every organ it samples; dGTEx confirms only the tissues it powers adequately for the scramble null (lung, muscle, adipose); and any cell that fails the ortholog-scramble null at *p<0.05* is reported as suggestive and relegated to Supplementary Table S5 with its *n*, without re-routing it to a different substrate post hoc. Target expression matrices were $\log _2$-transformed and restricted to the shared one-to-one ortholog set before projection.

#### Anatomical brain definition

Because target atlases (such as the Cardoso-Moreira dataset) define “brain” anatomically as the forebrain (cerebrum minus cerebellum), the pig brain model aggregates the corresponding PigGTEx forebrain subregions (Brain, frontal and cerebral cortex, frontal lobe, cerebrum, hippocampus, amygdala and striatum; *n=170* across four well-sampled stages), excluding cerebellum, hypothalamus, pituitary and peripheral ganglia to ensure anatomical consistency across species.

#### Determinants of transferability

Across the *n=40* pig-to-target cells of the Cardoso-Moreira panel (six organs *×* up to seven species), we tested three pre-specified hypotheses. H1 (phylogeny): Spearman correlation between species divergence time from the pig (TimeTree [71]) and transfer *ρ*. H2 (tissue): mean *ρ* per organ, and a control test of whether organ transcriptome complexity (per-organ number of expressed pig orthologs) explains the organ ranking (Spearman). H3 (sample-size confound): Spearman between target sample size and *ρ*, pooled and within organ. To weigh the two axes directly we additionally (i) regressed transfer *ρ* on organ identity and divergence time (Type-II ANOVA and df-adjusted unique $R^2$), benchmarking organ against a 5,000-permutation random six-level-factor null; (ii) computed the species-level Spearman between each species’ mean transfer and its divergence time (*n=7*), which avoids the pseudoreplication of the 40-cell test (the five eutherian species share one divergence bin); and (iii) fit a mixed model $\rho \sim$ divergence $+\,(1\!\mid \!\text {organ}) + (1\!\mid \!\text {species})$ to respect the crossed, organ-unbalanced design. Reported statistics are computed directly from the per-cell transfer values (Supplementary Table S5).

### Within-project batch validation

To assess whether cross-project technical variation confounds developmental signals, we performed a within-project fold-change validation. We identified the two largest single-project muscle cohorts that each span multiple developmental stages under uniform protocols: PRJNA488311 ($n_{\text {total}} = 81$) and PRJNA486202 ($n_{\text {total}} = 74$). Both cohorts predominantly sample the neonatal stage and span the neonatal-to-pre-pubertal window. We computed fold-changes across three developmental contrasts, all using neonatal as the baseline: neonatal vs. suckling, neonatal vs. juvenile, and neonatal vs. pre-pubertal. For each gene and each comparison, we computed $\log _2$ fold change (later stage/neonatal) and Mann–Whitney *U* *p*-value, with Benjamini–Hochberg FDR correction. We then correlated within-project fold-changes with full-dataset fold-changes using Pearson *r* genome-wide. A pooled within-project estimate was computed by averaging the fold-changes from both projects before correlation. Bootstrap confidence intervals (1,000 iterations) were computed for the pooled correlation at each comparison (Supplementary Table S2).

## Supplementary Information

Supplementary Material 1.

## Data Availability

All datasets analysed in this study are publicly available. The porcine postnatal training data were obtained from the PigGTEx consortium resource (https://piggtex.farmgtex.org/). The cross-species transfer analysis used two independent developmental atlases: the seven-species developmental transcriptomic atlas of Cardoso-Moreira et al. is available at ArrayExpress under accession E-MTAB-6814 (https://www.ebi.ac.uk/biostudies/studies/E-MTAB-6814) and via the EvoDevoApp portal (https://apps.kaessmannlab.org/evodevoapp/); the human developmental Genotype-Tissue Expression (dGTEx) project data are available via the GTEx Portal (https://gtexportal.org/).
