# Supplementary material for: Tissue identity is the dominant determinant of cross-species transferability of a porcine developmental programme
Source: BMC Genomics. 2026 Jul 4;27:919. doi: 10.1186/s12864-026-13103-0 (PMC13737508; doi:10.1186/s12864-026-13103-0)
Supplement: Supplementary file 1 [file 12864_2026_13103_MOESM1_ESM.pdf]

Supplementary Information

Tissue identity is the dominant determinant of cross-species transferability of a porcine developmental programme

Tianyuan Liu, Rujing Lei, Ilyas M. Khan, Peter Theobald

Contents

Supplementary Figures 1

Supplementary Tables 6

Supplementary Figures

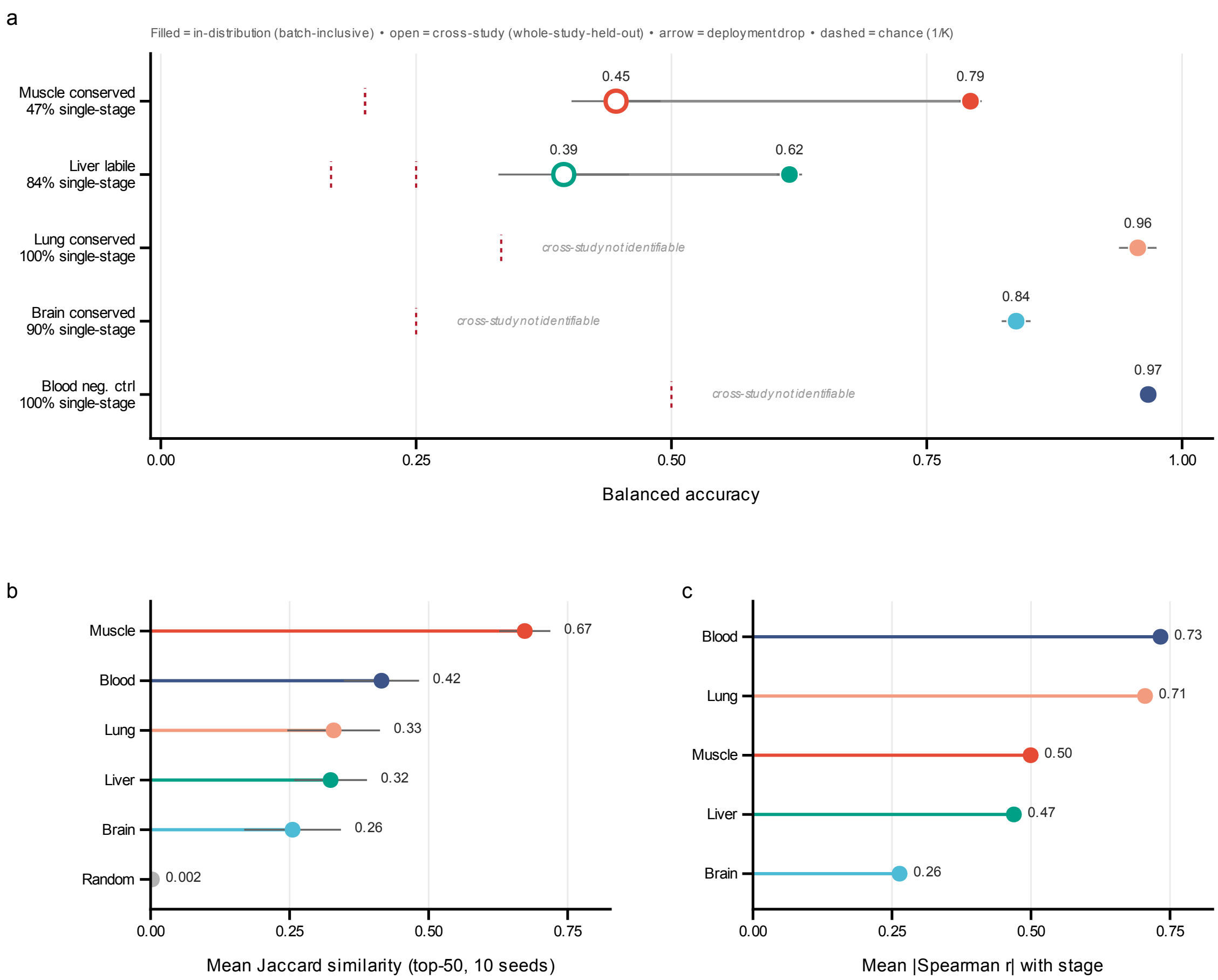

Supplementary Figure S1: Extended model evaluation: in-distribution versus cross-study staging accuracy, feature-selection stability, and feature–stage correlation. **a**, Within-pig staging balanced accuracy (BA) per tissue, drawn as a dumbbell: a filled marker for in-distribution (stratified, batch-inclusive) separability and, for the two tissues whose stages span multiple studies, an open marker for the cross-study (grouped, whole-study-held-out) deployment estimate, joined by an arrow tracing the deployment drop (muscle 0.79 to 0.45; liver 0.62 to 0.39, the latter over liver's four study-spanning stages; Supplementary Table S1). Markers are the mean and whiskers the s.d. across five random seeds; short red dashes mark each estimate's chance level ($1/K$, which differs between liver's six-stage in-distribution and four-stage cross-study evaluations). Tissues are ordered by their single-stage-study fraction (beside each label); for the three 100%-single-stage tissues the cross-study estimate is not identifiable. The conservation status beside each tissue label summarises cross-species transfer as a graded axis, not a pass/fail test: muscle, lung and brain transfer to independent atlases (conserved); liver transfers weakly, consistent with a labile developmental programme rather than a staging failure; and blood, which has no cross-species substrate, serves as a sex-confound negative control. **b**, Feature-selection stability (mean Jaccard similarity of the top 50 VIP features across ten seeds), shown as lollipops with s.d. whiskers; the grey *Random* point is the analytical random-selection expectation. **c**, Mean absolute Spearman correlation between the top 50 VIP features and developmental stage per tissue.

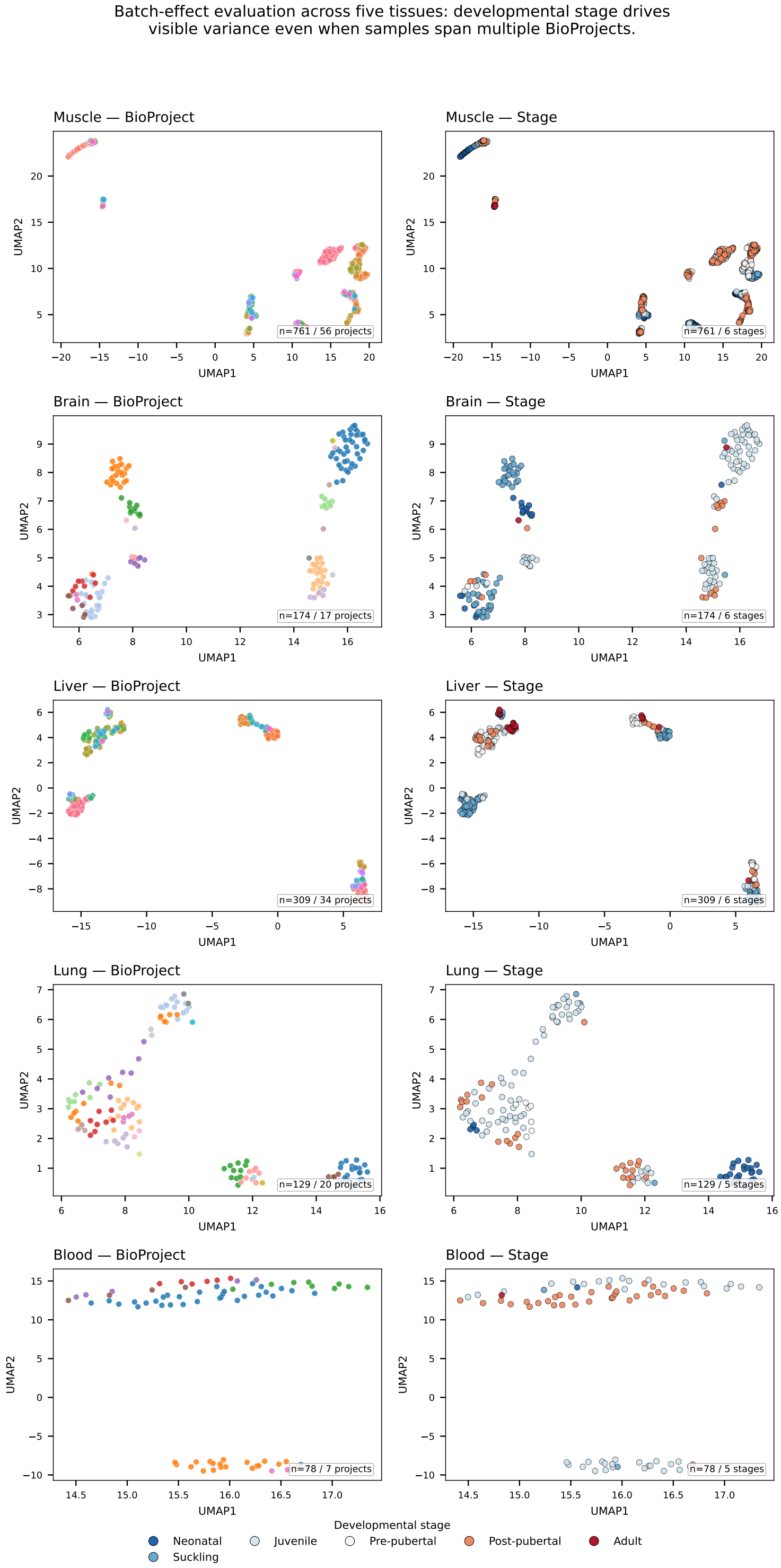

Supplementary Figure S2: Batch-effect evaluation across five tissues: developmental stage drives visible variance even when samples span multiple BioProjects. UMAP embeddings of the top 5,000 most-variable genes, with tissues arranged in rows. Left column shows samples coloured by BioProject; right column shows samples coloured by developmental stage. Sample sizes (n) and BioProject counts are annotated. UMAP parameters: `n_neighbors=15`, `min_dist=0.1`, euclidean metric, `random_state=42`, 10 PCA components.

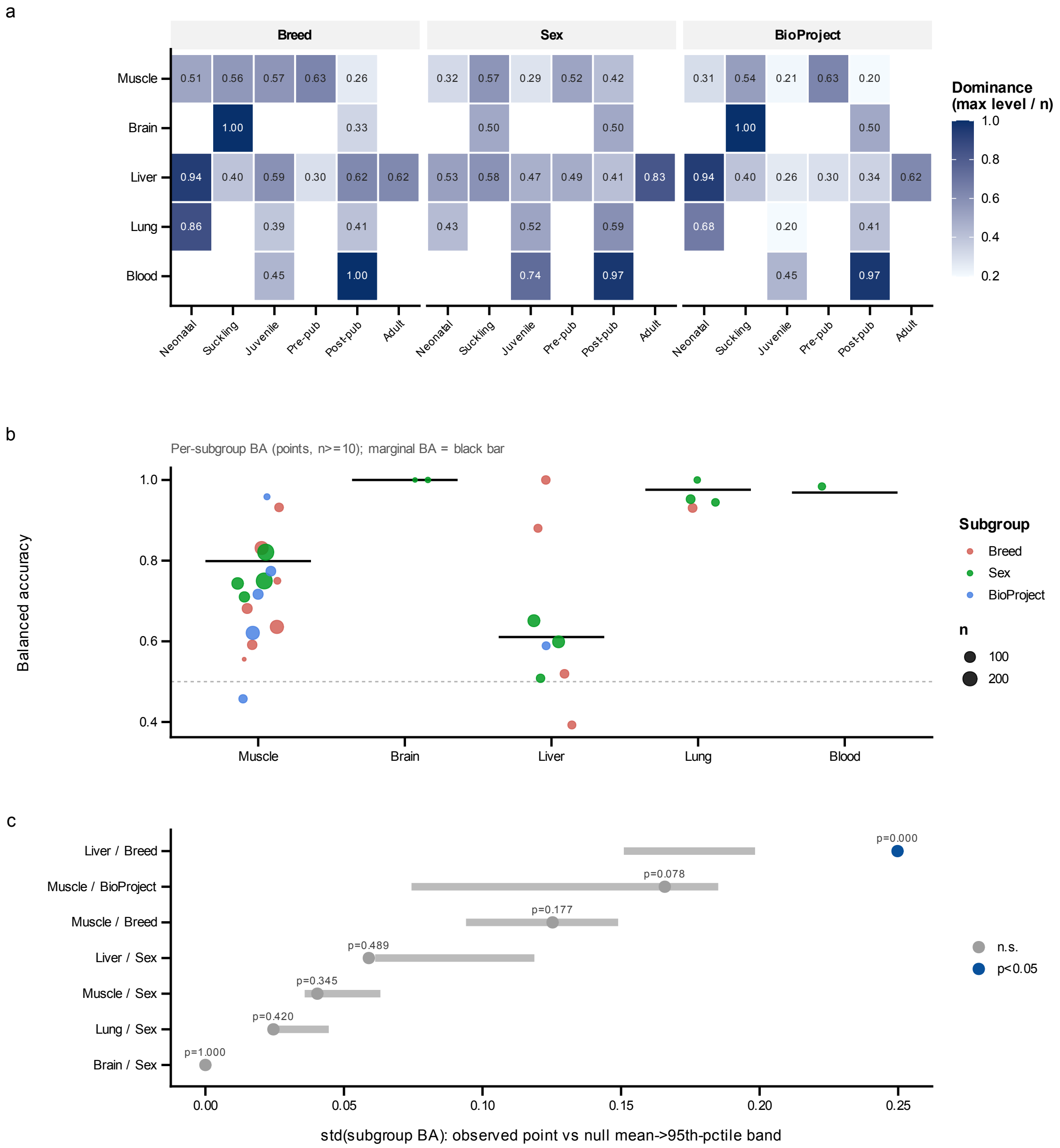

Supplementary Figure S3: Within-stage demographic heterogeneity and subgroup-sensitivity of the staging models. **a**, Within-stage dominance heatmaps (the fraction of samples in the single most-common level, max level / n; values in $[0.2, 1]$) across tissues and developmental stages stratified by Breed, Sex, and BioProject. **b**, Balanced accuracy (BA) per stratum (for subgroups with $n \geq 10$). The solid black bar per tissue marks the tissue's marginal BA; the dashed grey line is the 0.5 chance reference. **c**, Empirical null distribution of the standard deviation of BA from 1,000 within-stage permutations versus the observed standard deviation for a representative tissue.

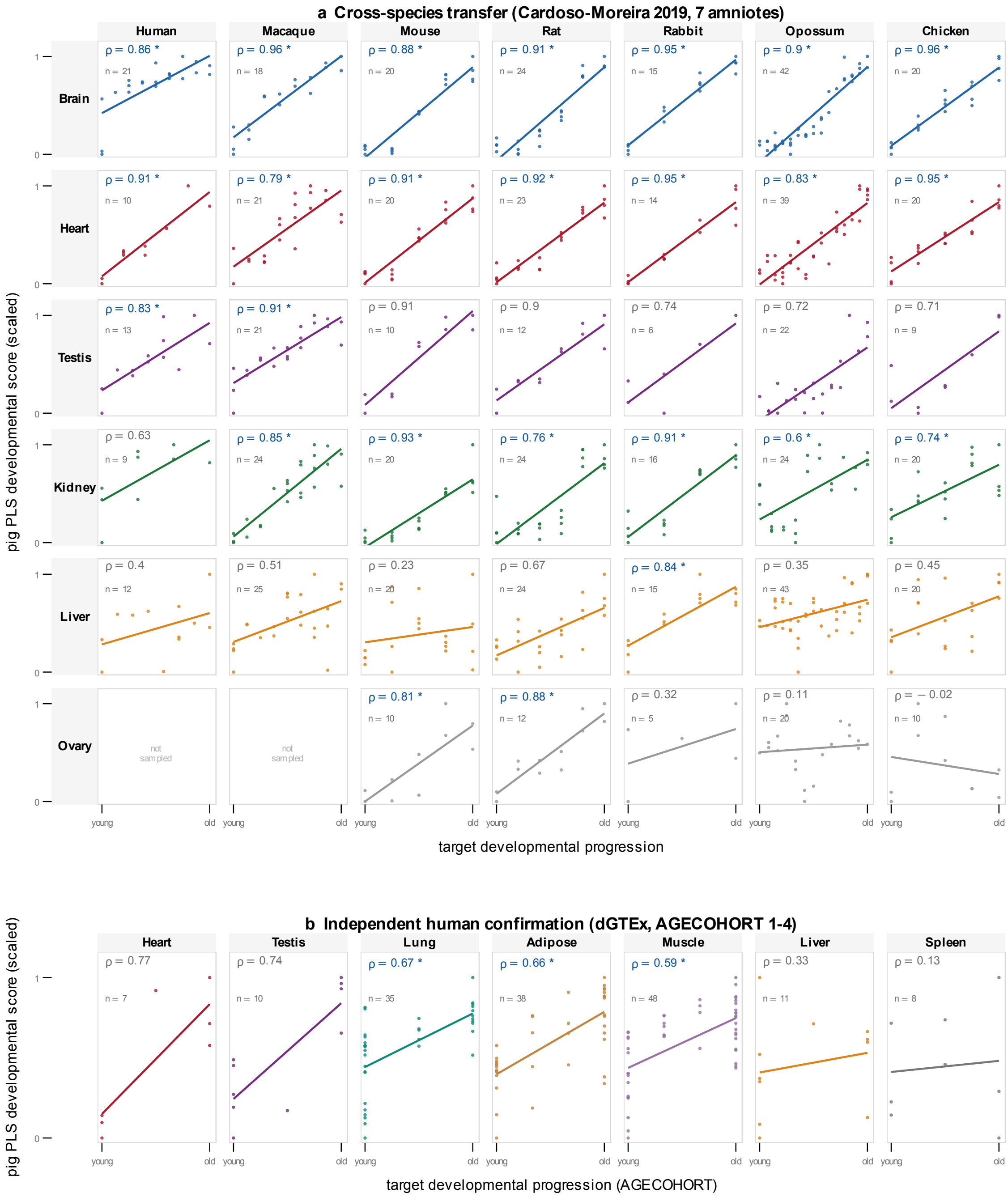

Supplementary Figure S4: Individual cross-species transfer correlation scatters. Each panel plots the pig-trained PLS developmental score projected onto target samples (y-axis) against the target's developmental stage ordering (x-axis). Both axes are min-max scaled for visualization. Bold blue values with asterisks denote cells passing the 1,000× ortholog-scramble null ($p < 0.05$); grey values denote non-significant cells; n is target sample size. Solid lines represent linear regression fits. **a**, Seven-species Cardoso-Moreira atlas (six organs × seven species). Blank cells indicate tissues not sampled in that species. Brain panels use the forebrain-aggregated pig construct. **b**, Human dGTEx developmental cohort (seven organs).

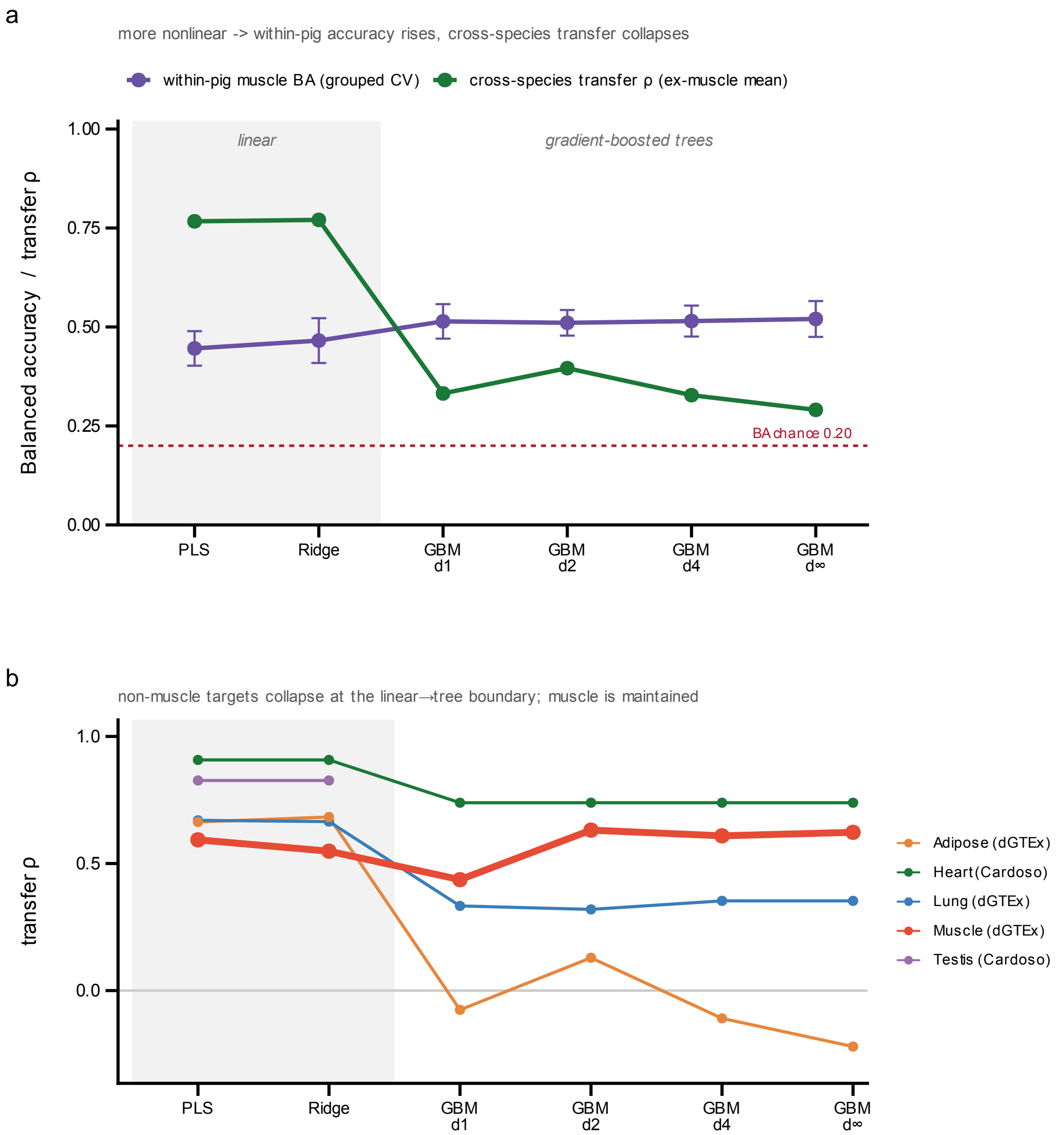

Supplementary Figure S5: The accuracy–transferability trade-off selects the linear model. **a**, Within-pig muscle balanced accuracy (grouped CV) and average cross-species transfer correlation ρ (non-muscle targets) across six staging estimators of varying nonlinearity: linear models (PLS, ridge) and trees (LightGBM depths 1, 2, 4, and unlimited). **b**, Individual cross-species transfer correlation ρ per target tissue (muscle, adipose, lung, heart, testis) across the six estimators, highlighting the loss of transferability in non-muscle targets under nonlinear tree-based models. The estimators and their compute footprint are listed in Supplementary Table S6.

Supplementary Tables

Supplementary Table S1: Within-pig staging: stage–study composition, in-distribution separability, and the cross-study deployment estimate. For each tissue we list the developmental stages retained under the drop-small policy, the sample size n, the number of contributing BioProjects, and the fraction of samples drawn from *single-stage* studies (the stage–study composition). The *in-distribution* balanced accuracy is from stratified cross-validation, which places samples from the same BioProject in both the training and test folds; it is therefore a within-cohort, **batch-inclusive** measure of stage separability and **not** a generalisation or deployment estimate. A valid *cross-study* deployment estimate requires holding out whole studies. This is identifiable for two tissues. For **muscle**, the tissue with substantial multi-stage studies (47% of samples from single-stage studies), it gives balanced accuracy 0.45 ± 0.04 (grouped cross-validation holding out whole studies, `StratifiedGroupKFold` over the muscle BioProjects, 5 folds $\times$ 5 seeds; ordinal $\rho = 0.66$; a ridge comparator gives 0.46, consistent), using all five retained stages. For **liver** it is also identifiable, over its four multi-cohort stages ($0.39^{\dagger}$). For the remaining three tissues (lung, blood, and brain; 100% single-stage studies) stage is perfectly confounded with BioProject, so a held-out study presents a developmental stage essentially unseen in training and the cross-study quantity is **not identifiable**; we do not report one there. The muscle in-distribution-versus-cross-study gap (from 0.79 to 0.45) shows how large batch-inclusive inflation can be where a clean comparator exists.

Tissue	Stages	n	BioProj.	Single-stage %	In-dist. BA	Cross-study BA
Muscle	5	757	54	47	0.793	**0.45**
Lung	3	119	17	100	0.957	—
Liver	6	309	34	84	0.616	$0.39^{\dagger}$
Blood	2	74	7	100	0.967	—
Brain	4	170	17	90	0.838	—

Single-stage %: percentage of retained samples from single-stage BioProjects. In-dist. BA: in-distribution (stratified, batch-inclusive) balanced accuracy, a within-cohort separability measure, *not* a deployment estimate. Cross-study BA: grouped-CV (whole-study-held-out) deployment estimate; — denotes *not identifiable* (a held-out study presents a developmental stage absent from training). Chance is $1/K$ for K stages. The counts here are the stage-pruned staging subset; their reconciliation with the pre-drop embedding superset (and the five-tissue total of 1,429 staged samples) is given in Supplementary Table S3. † Liver's cross-study BA is computed over the four stages present in at least two BioProjects (suckling, juvenile, pre-pubertal, and post-pubertal; balanced accuracy 0.39 ± 0.06, ordinal $\rho = 0.44$, chance 0.25); its neonatal and adult stages are each confined to a single dominant BioProject (Methods). Muscle's estimate uses all five retained stages.

Supplementary Table S2: Within-project fold-change validation across developmental contrasts. Developmental fold-changes ($\log_2$ scale) were computed within single-project muscle cohorts, each spanning the neonatal-to-pre-pubertal window under uniform laboratory protocols. These were correlated with full-dataset estimates across three stage comparisons (all using neonatal as baseline), over all 31,908 expressed genes. Pooled within-project fold-changes were computed by averaging across both projects before correlating with full-dataset estimates. CI = 95% bootstrap confidence interval (1,000 iterations). Per-project results are available in the analysis repository. Both within-project cohorts are predominantly neonatal, so neonatal serves as the contrast baseline; the muscle classifier's retained stages and the dropped adult samples are detailed in Supplementary Table S1 and Methods.

	Full dataset		Within-project			
Comparison	n_{Neo}	n_{later}	n_{Neo}	n_{later}	Pooled r [95% CI]	p-value
neonatal vs. suckling	156	87	95	11	0.772 [0.77–0.78]	$< 10^{-300}$
neonatal vs. juvenile	156	56	95	24	0.763 [0.75–0.77]	$< 10^{-300}$
neonatal vs. pre-pubertal	156	227	95	20	0.799 [0.79–0.81]	$< 10^{-300}$

Supplementary Table S3: Variance decomposition of PC1–PC5 with respect to developmental stage and BioProject across five tissues. For each tissue we projected the $\log_2(\mathrm{TPM}+1)$ values of the top 5,000 most-variable genes into a 10-component PCA. For each principal component we fit ordinary-least-squares models PC $\sim$ Stage, PC $\sim$ BioProject, and the combined PC $\sim$ Stage + BioProject, and report the coefficient of determination (R^2). n_{samples} is the number of samples per tissue available after merging metadata; n_{projects} is the number of distinct BioProjects represented. These counts are the **pre-drop superset** (every sample entering the embedding, including those in stages later removed by the drop-small policy), so they exceed the stage-pruned n used for staging in Supplementary Table S1 (Muscle 761 vs. 757, Lung 129 vs. 119, Blood 78 vs. 74, Brain 174 vs. 170; Liver 309 in both). Each difference is the count of samples in dropped sub-threshold stages (for muscle, the 4 adult samples). The few extra BioProjects here (Muscle +2, Lung +3, Brain +0) are studies contributing only to those dropped stages. The five-tissue funnel is therefore 1,874 samples with a parseable developmental stage (Fig. 1c), narrowing to 1,451 in this embedding superset and 1,429 in the staged subset of Table S1. BioProject carries the larger absolute variance share in every tissue because most PigGTEx projects sample a single narrow age window, the stage–study confound in direct form. Nonetheless stage alone still explains a substantial fraction of early-component variance in muscle (PC1 0.48) and brain (PC1 0.47), recovering only at later components in liver and blood.

Tissue	PC	R^2_{stage}	$R^2_{\text{bioproject}}$	R^2_{combined}	n_{projects}	n_{samples}
Muscle	PC1	0.484	0.803	0.895	56	761
Muscle	PC2	0.311	0.750	0.811	56	761
Muscle	PC3	0.135	0.862	0.886	56	761
Muscle	PC4	0.208	0.718	0.821	56	761
Muscle	PC5	0.034	0.846	0.852	56	761
Brain	PC1	0.468	0.942	0.942	17	174
Brain	PC2	0.121	0.982	0.983	17	174
Brain	PC3	0.142	0.980	0.980	17	174
Brain	PC4	0.147	0.978	0.978	17	174
Brain	PC5	0.391	0.969	0.979	17	174
Liver	PC1	0.146	0.935	0.936	34	309
Liver	PC2	0.178	0.962	0.962	34	309
Liver	PC3	0.103	0.919	0.920	34	309
Liver	PC4	0.120	0.869	0.881	34	309
Liver	PC5	0.385	0.641	0.751	34	309
Lung	PC1	0.279	0.881	0.882	20	129
Lung	PC2	0.557	0.895	0.895	20	129
Lung	PC3	0.285	0.813	0.814	20	129
Lung	PC4	0.121	0.752	0.753	20	129
Lung	PC5	0.259	0.904	0.911	20	129
Blood	PC1	0.023	0.806	0.817	7	78
Blood	PC2	0.359	0.954	0.960	7	78
Blood	PC3	0.316	0.727	0.748	7	78
Blood	PC4	0.112	0.794	0.815	7	78
Blood	PC5	0.347	0.914	0.934	7	78

Supplementary Table S4: Top 20 developmental-stage biomarkers per tissue (PLS-VIP). Genes ranked by Variable Importance in Projection (VIP) from each tissue's final ordinal partial-least-squares model (OrdinalPLS, 6-stage); VIP>1 flags above-average contribution (mean $VIP^2 = 1$). Biomarkers are read directly from the model weights, not from a separate statistical test. "Dir." is the sign of the PLS regression coefficient onto the ordinal stage index: + accelerator (expression rises toward later stage), − decelerator (falls toward later stage). Symbols are from mygene.info (pig) / UniProt; functional descriptions are online-verified against UniProt human-reviewed Swiss-Prot and PubMed. Loci with no curated function are labelled uncharacterised/novel. "Peak" is the stage with the highest mean $\log_2$(TPM+1) among retained stages. *Only Muscle is leakage-robust under grouped cross-validation; for Brain/Blood/Lung/Liver some top-VIP genes may track stage confounded with study (see main text).*

Tissue	Rk	Gene	Dir.	VIP	Function (online-verified)	Peak
Muscle	1	*MSS51*	+	2.56	ZMYND17/MSS51; skeletal-muscle mitochondrial regulator and target of myostatin/TGF-beta signalling that controls fatty-acid oxidation, glycolysis and oxidative phosphorylation. [1]	Pre-pubertal
	2	*CPLX1*	−	2.53	Complexin-1; positively regulates a late step of SNARE-mediated vesicle exocytosis (synaptic vesicles). [2]	Neonatal
	3	*ENSSSCG00000036741*	−	2.53	Unannotated/novel pig locus (no current Ensembl annotation)	Suckling
	4	*GKAP1*	+	2.51	G-kinase anchoring protein 1; modulates IRS1 tyrosine phosphorylation and insulin signalling. [3]	Post-pubertal
	5	*ASB14*	+	2.51	Ankyrin-repeat and SOCS-box protein 14; substrate-recognition subunit of an ECS (Elongin-Cullin-SOCS) E3 ubiquitin ligase complex. [4]	Pre-pubertal
	6	*HOXC8*	+	2.45	Sequence-specific homeobox transcription factor providing positional/segmental identity along the anteroposterior axis; involved in muscle and skeletal development. [5]	Pre-pubertal
	7	*FGFRL1*	−	2.44	Fibroblast growth factor receptor-like 1; FGFR-family receptor that binds FGF ligands and heparin but lacks the intracellular tyrosine-kinase domain, exerting a negative effect on cell proliferation; required for diaphragm/slow-muscle-fibre development. [6, 7]	Neonatal
	8	*TEX9*	+	2.44	Testis-expressed 9; poorly characterised, no curated FUNCTION; treat as uncharacterised.	Post-pubertal
	9	*OPTN*	+	2.42	Optineurin; Golgi maintenance, membrane trafficking, exocytosis and autophagy adaptor (myosin VI/Rab8). [8]	Pre-pubertal
	10	*DLK1*	−	2.42	Delta-like non-canonical Notch ligand 1 (Pref-1); imprinted, paternally expressed transmembrane EGF-repeat regulator of cell differentiation that gates myoblast proliferation and myogenic differentiation. [9]	Neonatal
	11	*PGM2L1*	+	2.42	Glucose-1,6-bisphosphate synthase; produces the allosteric regulator glucose-1,6-bisphosphate; central carbohydrate metabolism. [10]	Pre-pubertal
	12	*CCDC160*	+	2.39	Coiled-coil domain containing 160; uncharacterised.	Post-pubertal
	13	*LOC110257362*	−	2.37	uncharacterised	Neonatal
	14	*ENSSSCG00000043913*	+	2.36	Unannotated/novel pig locus (no current Ensembl annotation)	Pre-pubertal
	15	*DDX25*	+	2.35	ATP-dependent DEAD-box RNA helicase (GRTH); component of messenger ribonucleoprotein particles that transports target mRNAs and regulates their translation; characterised in testicular germ-cell development. [11]	Pre-pubertal
	16	*HS3ST5*	+	2.35	Heparan sulfate-glucosamine 3-O-sulfotransferase 5; modifies heparan sulfate chains. [12]	Pre-pubertal
	17	*LOC100524609*	+	2.33	uncharacterised	Pre-pubertal
	18	*ENSSSCG00000050649*	−	2.33	Unannotated/novel pig locus (no current Ensembl annotation)	Neonatal
	19	*NEDD4*	+	2.33	E3 ubiquitin-protein ligase; regulates IGF-1/insulin signalling and is required for normal muscle/neuromuscular development. [13]	Post-pubertal
	20	*TTLL7*	+	2.33	Tubulin polyglutamylase TTLL7; adds polyglutamate side chains preferentially to beta-tubulin C-terminal tails; required for neurite growth, with beta-tubulin polyglutamylation rising during postnatal neuronal maturation. [14]	Pre-pubertal
Brain	1	*CARMIL3*	−	2.60	Capping-protein regulator and myosin-1 linker 3 (CARMIL3/LRRC16B); regulates actin filament capping during cytoskeleton-mediated synaptic maturation. [15]	Suckling
	2	*ASF1B*	−	2.56	Anti-silencing function 1B histone chaperone; replication-coupled nucleosome assembly; proliferation marker. [16]	Suckling
	3	*ENSSSCG00000031180*	−	2.53	Unannotated/novel pig locus (no current Ensembl annotation)	Suckling
	4	*CDC20*	−	2.49	Cell-division-cycle 20; substrate-specific activator of the anaphase-promoting complex (APC/C); drives mitotic progression. [17]	Suckling
	5	*ENSSSCG00000048208*	−	2.47	Unannotated/novel pig locus (no current Ensembl annotation)	Suckling

continued on next page

Table S4 continued from previous page

Tissue	Rk	Gene	Dir.	VIP	Function (online-verified)	Peak
	6	*PIMREG*	−	2.46	PICALM-interacting mitotic regulator; controls metaphase-to-anaphase transition (mitosis). [18]	Suckling
	7	*CDCA3*	−	2.46	Cell division cycle-associated protein 3 (Tome-1); F-box-like trigger of mitotic entry; substrate-recognition subunit of an SCF/Skp1 E3 ligase that ubiquitinates and degrades WEE1 kinase at the G2/M transition. [19]	Suckling
	8	*KLHL14*	−	2.45	Kelch-like protein 14; BTB-Kelch substrate-adaptor expressed by corticospinal neuron subpopulations during cortical development, where it restricts axon extension to bulbar-cervical segments. [20]	Suckling
	9	*ENSSSCG00000049082*	−	2.44	Unannotated/novel pig locus (no current Ensembl annotation)	Suckling
	10	*SIPA1L3*	−	2.44	Signal-induced proliferation-associated 1-like 3; epithelial morphogenesis, polarity, adhesion and cytoskeleton. [21]	Suckling
	11	*TBX6*	−	2.43	T-box transcription factor 6; sequence-specific transcription factor specifying paraxial mesoderm by repressing the Sox2 N1 enhancer to suppress neural fate in axial stem cells; expressed transiently in early development. [22]	Suckling
	12	*PTH1R*	−	2.43	Parathyroid hormone/parathyroid hormone-related peptide receptor; G-protein-coupled receptor signalling via adenylate cyclase/cAMP that drives osteoblast differentiation and, in the growth plate, sets the timing of chondrocyte differentiation. [23, 24]	Suckling
	13	*UBE2C*	−	2.43	Ubiquitin-conjugating enzyme E2 C (UbcH10); APC/C-associated E2 that initiates Lys-11-linked polyubiquitin chains to destroy mitotic cyclins and securin and drive mitotic exit. [25, 26]	Suckling
	14	*PTTG1*	−	2.43	Securin (PTTG1); anaphase inhibitor that restrains separase (ESPL1) until its APC/C-driven destruction permits sister-chromatid separation. [27]	Suckling
	15	*MAMSTR*	−	2.42	MEF2-activating motif and SAP domain-containing transcriptional regulator (MASTR); transcriptional coactivator that stimulates MEF2 and cooperates with the myogenic factor MyoD to promote skeletal-muscle gene expression. [28, 29]	Suckling
	16	*NEUROD6*	−	2.42	Neuronal differentiation factor 6 (NEX/Math2); NeuroD-family basic helix-loop-helix transcription factor; binds E-box motifs as an E-protein heterodimer to drive neuronal differentiation in the developing CNS. [30]	Suckling
	17	*TROAP*	−	2.42	Tastin (trophinin-associated protein); maintains mitotic spindle bipolarity and centrosome integrity; protein level peaks in G2/M and falls after cell division, marking proliferating/cycling cells. [31]	Suckling
	18	*GNB3*	−	2.41	Guanine nucleotide-binding protein G(I)/G(S)/G(T) subunit beta-3 (GNB3); beta subunit of heterotrimeric G proteins transducing GPCR signals; forms the beta subunit of cone transducin in retinal phototransduction. [32]	Suckling
	19	*B3GNT7*	−	2.41	uncharacterised	Suckling
	20	*PLXNB3*	−	2.41	Plexin-B3; receptor for semaphorin SEMA5A that regulates cell migration. [33]	Suckling
Liver	1	*CXCL14*	+	2.92	C-X-C motif chemokine ligand 14; chemoattractant; homeostatic chemokine implicated in metabolism and immune surveillance. [34]	Adult
	2	*CD320*	−	2.89	CD320 / transcobalamin receptor; mediates cellular cobalamin (vitamin B12) uptake. [35]	Neonatal
	3	*ALDH18A1*	−	2.86	Delta-1-pyrroline-5-carboxylate synthase; bifunctional enzyme in proline/ornithine/arginine biosynthesis from glutamate. [36]	Neonatal
	4	*LOC102164754*	+	2.81	uncharacterised	Adult
	5	*ID1*	−	2.81	Inhibitor of DNA-binding 1; dominant-negative regulator of basic helix-loop-helix transcription factors that restrains differentiation and promotes proliferation. [37, 38]	Neonatal
	6	*MKRN2OS*	−	2.80	uncharacterised	Neonatal
	7	*CRABP1*	−	2.79	Cellular retinoic-acid-binding protein 1; sequesters retinoic acid in the cytoplasm and regulates its availability to nuclear retinoic-acid receptors. [39]	Neonatal
	8	*GLRA3*	+	2.70	Glycine receptor alpha-3 subunit; glycine-gated chloride channel. [40]	Adult
	9	*MUC13*	+	2.65	Mucin 13; epithelial transmembrane mucin in cell signalling and protection. [41]	Post-pubertal
	10	*ENSSSCG00000013714*	+	2.64	SEA domain-containing protein (uncharacterised)	Post-pubertal
	11	*ENSSSCG00000050649*	−	2.61	Unannotated/novel pig locus (no current Ensembl annotation)	Neonatal
	12	*RAB34*	−	2.59	Ras-related protein Rab-34; Golgi-associated Rab GTPase regulating intracellular membrane trafficking and the redistribution of lysosomes to the peri-Golgi region via RILP. [42]	Neonatal
	13	*TAFA5*	+	2.59	TAFA chemokine-like family member 5 (FAM19A5); regulates cell proliferation/migration via GPCRs. [43]	Adult

continued on next page

Table S4 continued from previous page

Tissue	Rk	Gene	Dir.	VIP	Function (online-verified)	Peak
	14	*ORM1*	−	2.59	Alpha-1-acid glycoprotein 1 (orosomucoid-1); liver-secreted plasma transport/drug-binding glycoprotein and immunomodulator; in pig a negative acute-phase protein, highest in neonatal liver and falling toward adulthood. [44]	Neonatal
	15	*ELN*	−	2.59	Elastin (tropoelastin); core structural protein of extracellular-matrix elastic fibres, conferring elasticity and recoil; transcriptionally activated in fetal/perinatal life and declining postnatally. [45]	Neonatal
	16	*ATP5MJ*	−	2.58	ATP synthase membrane subunit j (6.8 kDa proteolipid, C14orf2); small integral-membrane component of the Fo sector of mitochondrial F1Fo ATP synthase (complex V). [46]	Neonatal
	17	*RIMS1*	+	2.58	Regulating synaptic membrane exocytosis 1; Rab effector / active-zone scaffold for neurotransmitter release. [47]	Post-pubertal
	18	*ENSSSCG00000003318*	−	2.57	Unannotated/novel pig locus (no current Ensembl annotation)	Suckling
	19	*ENSSSCG00000038150*	+	2.55	Long non-coding RNA (uncharacterised)	Adult
	20	*ENSSSCG00000038539*	+	2.55	Unannotated/novel pig locus (no current Ensembl annotation)	Adult
Blood	1	*ENSSSCG00000033734*	−	3.26	Unannotated/novel pig locus (no current Ensembl annotation)	Juvenile
	2	*LOC110261493*	+	3.17	uncharacterised	Post-pubertal
	3	*ENSSSCG00000012178*	−	3.12	Eukaryotic translation initiation factor 2 subunit 3	Juvenile
	4	*ENSSSCG00000032661*	−	3.10	Oral-facial-digital syndrome 1 protein isoform 1	Juvenile
	5	*LOC110257883*	−	2.97	uncharacterised	Juvenile
	6	*ENSSSCG00000026430*	−	2.96	RNA helicase	Juvenile
	7	*ENSSSCG00000041163*	+	2.90	Long non-coding RNA (uncharacterised)	Post-pubertal
	8	*ENSSSCG00000027046*	−	2.87	ubiquitinyl hydrolase 1	Juvenile
	9	*CNKSR3*	+	2.87	Connector enhancer of kinase suppressor of Ras 3; regulates ENaC-mediated transepithelial sodium transport (aldosterone). [48]	Post-pubertal
	10	*ZFY*	−	2.84	Zinc-finger protein Y-linked; probable transcriptional activator on the Y chromosome (MALE-specific). [49]	Juvenile
	11	*PKP2*	+	2.83	Plakophilin-2; desmosomal cell-cell junction component. [50]	Post-pubertal
	12	*EIF1AY*	−	2.82	Eukaryotic translation initiation factor 1A Y-linked; 43S pre-initiation complex component (Y-chromosome paralogue, MALE-specific). [51]	Juvenile
	13	*TMEM11*	+	2.80	Transmembrane protein 11; mitochondrial morphogenesis. [52]	Post-pubertal
	14	*RPLP1*	+	2.79	60S acidic ribosomal protein P1; translation elongation. [53]	Post-pubertal
	15	*ACER2*	+	2.79	Alkaline ceramidase 2; Golgi ceramidase generating sphingosine; sphingolipid metabolism. [54]	Post-pubertal
	16	*ENSSSCG00000044599*	+	2.77	Unannotated/novel pig locus (no current Ensembl annotation)	Post-pubertal
	17	*ENSSSCG00000031608*	+	2.75	Unannotated/novel pig locus (no current Ensembl annotation)	Post-pubertal
	18	*FAM110B*	+	2.75	Family with sequence similarity 110 member B; centrosome-associated protein implicated in G1 cell-cycle progression (poorly characterised). [55]	Post-pubertal
	19	*ENSSSCG00000031003*	+	2.74	Uncharacterized protein (uncharacterised)	Post-pubertal
	20	*ENSSSCG00000040651*	−	2.74	Unannotated/novel pig locus (no current Ensembl annotation)	Juvenile
Lung	1	*ENSSSCG00000031037*	+	3.25	Immunoglobulin gene segment (Ig-like; not individually characterised)	Post-pubertal
	2	*ENSSSCG00000041880*	+	3.22	Immunoglobulin gene segment (Ig-like; not individually characterised)	Post-pubertal
	3	*ENSSSCG00000048391*	+	3.19	Immunoglobulin gene segment (Ig-like; not individually characterised)	Post-pubertal
	4	*ENSSSCG00000010044*	+	3.18	Immunoglobulin gene segment (Ig-like; not individually characterised)	Post-pubertal
	5	*ENSSSCG00000033640*	+	3.16	Immunoglobulin gene segment (Ig-like; not individually characterised)	Post-pubertal
	6	*ENSSSCG00000008769*	+	3.13	*CD68* (macrosialin); macrophage marker. [56]	Post-pubertal
	7	*JCHAIN*	+	3.11	Joining chain of multimeric IgA and IgM; links Ig monomers; plasma-cell / mucosal antibody marker. [57]	Post-pubertal
	8	*ENSSSCG00000032237*	+	3.10	Immunoglobulin gene segment (Ig-like; not individually characterised)	Post-pubertal
	9	*ENSSSCG00000035443*	+	3.09	Immunoglobulin gene segment (Ig-like; not individually characterised)	Post-pubertal
	10	*CD6*	+	3.05	CD6; T-cell adhesion molecule, binds ALCAM/CD166, regulates T-cell responses. [58]	Post-pubertal
	11	*CD8A*	+	3.04	CD8 alpha chain; cytotoxic-T-lymphocyte co-receptor; adaptive immunity marker. [59]	Post-pubertal
	12	*ENSSSCG00000044779*	+	3.03	Immunoglobulin gene segment (Ig-like; not individually characterised)	Post-pubertal
	13	*CD3E*	+	3.02	CD3 epsilon; component of the TCR-CD3 complex; pan-T-cell marker essential for adaptive immune response. [60]	Post-pubertal

continued on next page

Table S4 continued from previous page

Tissue	Rk	Gene	Dir.	VIP	Function (online-verified)	Peak
	14	*ENSSSCG00000036983*	+	3.00	Immunoglobulin gene segment (Ig-like; not individually characterised)	Post-pubertal
	15	*FBN3*	−	3.00	Fibrillin-3; structural glycoprotein of extracellular calcium-binding microfibrils; most highly expressed in fetal tissues including developing lung, with low postnatal levels. [61]	Neonatal
	16	*IGF2BP1*	−	3.00	Insulin-like growth factor 2 mRNA-binding protein 1 (IMP1); oncofetal m6A-reader RNA-binding protein that stabilizes target transcripts such as ACTB and MYC. [62]	Neonatal
	17	*ENSSSCG00000045982*	+	2.98	Immunoglobulin gene segment (Ig-like; not individually characterised)	Post-pubertal
	18	*CCN5*	+	2.97	Cellular communication network factor 5 (WISP2); matricellular protein modulating adhesion and tissue turnover. [63]	Post-pubertal
	19	*SIT1*	+	2.96	Signalling threshold-regulating transmembrane adapter 1; negatively regulates T-cell-receptor (TCR) signalling and is involved in positive selection of T-cells (immune marker; matches the Lung T-cell context). [64]	Post-pubertal
	20	*CD3D*	+	2.95	CD3 delta; invariant subunit of the TCR-CD3 complex (CD3 delta-epsilon heterodimer) that drives complex assembly and ITAM-mediated signal transduction; pan-T-cell marker. [65]	Post-pubertal

Supplementary Table S5: Full per-tissue, per-species transfer matrix of the pig-trained developmental-stage model (ordinal PLS). A single ordinal partial-least-squares (PLS) model trained on pig developmental stages (six stages; day boundaries 3/28/90/165/540) was projected onto held-out cross-species developmental transcriptomes through one-to-one symbol orthologues, and transfer scored as the ordinal Spearman correlation (ρ) between projected and true developmental order. Significance is the empirical p from a 1000× ortholog-scramble null (permuting the gene↔gene correspondence). **(a)** dGTEx human transfer; because the deep human developmental cohorts are small for several organs, only the well-powered organs (Lung, Muscle, Adipose; each $n \geq 35$ and rejecting the scramble null at $p < 0.05$) are taken as confirmation, while Heart ($n = 7$), Testis ($n = 10$), Liver ($n = 11$) and Spleen ($n = 8$) are flagged underpowered and are not used for the heart/testis claims (which rest on the well-sampled Cardoso panel). **(b)** Cardoso *et al.* (2019) seven-species × six-organ matrix, per-organ mean ρ, and pig-to-species divergence (MYA). Ovary lacks Human and Macaque samples ($n = 5$ species); other organs span $n = 7$. n_{orth} is the number of one-to-one orthologues per species. **Pig** n (panel a) and **Pig train** n **(**K**)** (panel b) give the number of PigGTEx samples used to fit each organ's pig developmental model and the number K of retained stages (six-stage scheme, drop-small < 10 per stage); the brain model is the forebrain-aggregated construct ($n = 170$, four stages) and every other organ uses its single-label PigGTEx tissue.

(a) dGTEx human, per-organ transfer (PLS ρ).

Organ	Pig n	PLS ρ	n_{tgt}	scramble p	status
Heart	55	0.77	7	0.584	underpowered
Testis	48	0.74	10	0.253	underpowered
**Lung**	119	0.67	35	0.006	confirmed
**Adipose**	128	0.66	38	0.002	confirmed
**Muscle**	757	0.59	48	0.003	confirmed
Liver	309	0.33	11	0.467	underpowered
Spleen	56	0.13	8	0.816	underpowered

Confirmed (well-powered, scramble-significant): Lung, Adipose, Muscle.

(b) Cardoso *et al.* (2019), seven-species × six-organ transfer (PLS ρ).

	MYA	Organ (PLS ρ)						
Species	from pig	Brain	Heart	Kidney	Liver	Ovary	Testis	n_{orth}
Human	94	0.86	0.91	0.63	0.40	—	0.83	15,200
Macaque	94	0.96	0.79	0.85	0.51	—	0.91	12,219
Mouse	94	0.88	0.91	0.93	0.23	0.81	0.91	14,689
Rat	94	0.91	0.92	0.76	0.67	0.88	0.90	12,730
Rabbit	94	0.95	0.95	0.91	0.84	0.32	0.74	12,070
Opossum	159	0.90	0.83	0.60	0.35	0.11	0.72	11,284
Chicken	319	0.96	0.95	0.74	0.45	−0.02	0.71	5,951
**Per-organ mean**		0.92	0.89	0.77	0.49	0.42	0.82	
**Pig train** n **(**K**)**		170 (4)	55 (3)	32 (2)	309 (6)	69 (4)	48 (3)	

The deepest split, pig → chicken (319 MYA), retains strong Heart ($\rho = 0.95$) and Testis ($\rho = 0.71$) transfer; divergence time explains little of the variance (Spearman $\rho_s = -0.25$, $p = 0.11$ over $n = 40$ cells; a variance partition over the six-organ × seven-species design attributes a raw (unadjusted) unique $R^2 = 0.54$ to organ vs. 0.05 to divergence; the df-adjusted Type-II values reported in the main text are 0.49 and 0.04).

Model	Family	Stratified BA (leaky)	Grouped BA (honest; 0.20)	Order-aware?	Transfer-capable?	Biomarker?	Peak RAM (GB)	CPU-time
Ordinal GBM (Frank–Hall)	tree	0.860	0.537	yes	no	gain†	2.73	4h52m
PCA + ridge	linear*	0.753	0.515	yes	partial	weak	0.72	3m56s
Histogram GBM	tree	0.892	0.493	no	no	gain†	2.25	26h21m
Ridge (variance-5k)	linear	0.830	0.490	yes	yes	weak	–	1m32s
Linear SVC	linear	0.918	0.487	no	yes	weak	1.29	3h18m
Multiclass GBM	tree	0.885	0.484	no	no	gain†	2.95	16h12m
Ridge (continuous)	linear	0.865	0.465	yes	yes	weak	0.61	2m28s
Ridge (std-weighted)	linear	0.865	0.465	yes	yes	weak	1.00	2m47s
Gradient boosting	tree	0.854	0.462	no	no	gain†	0.92	42h04m
MLP	nonlinear	0.872	0.460	no	no	no	0.59	0m31s
Elastic-net logistic	linear	0.877	0.447	no	yes	weak	0.99	59m19s
Random forest	tree	0.846	0.444	no	no	gain†	0.95	25m56s
PLS-DA	linear	0.750	0.444	no	yes	VIP	1.45	20m20s
PCA + logistic	linear*	0.885	0.441	no	partial	weak	1.05	6m19s
Extra trees	tree	0.865	0.430	no	no	gain†	0.95	15m59s
**PLS (chosen)**	**linear**	**0.793**	**0.427**	**yes**	**yes**	**VIP**	**1.29**	**10m52s**
kNN	nonlinear	0.791	0.356	no	no	no	1.00	2m34s
Gaussian NB	nonlinear	0.389	0.316	no	no	no	0.93	1m35s
Chance ($1/K$)	—	0.201	0.20	—	—	—	–	–

Supplementary Table S6: Within-pig model selection: why PLS is the right model despite a modest honest accuracy. Eighteen classifiers benchmarked on muscle, the only tissue for which a cross-study test is identifiable, ranked by leakage-controlled within-pig staging accuracy: macro balanced accuracy under `StratifiedGroupKFold` on BioProject (whole studies held out), scikit-learn defaults with no per-fold tuning, mean over 5 seeds; chance = $1/K = 0.20$ (5 kept muscle stages). **Every model's apparent within-pig accuracy is inflated by batch leakage.** Under ordinary stratified cross-validation, which lets samples from one study fall in both the training and test folds, all estimators bar the degenerate Gaussian NB look strong (*Stratified BA*, 0.75–0.92); holding out whole studies collapses them to 0.36–0.54 (*Grouped BA*), a +0.24 to +0.44 BA inflation that is universal rather than specific to any one model. This is why the high within-pig number is never quoted as a generalisation estimate (Methods; main Fig. 3). The single most accurate honest stager is a gradient-boosted tree (ordinal GBM, 0.537), but trees cannot transfer across species (see *Transfer-capable?* below; Methods; Fig. S5). **Order-aware?** balanced accuracy is order-blind—it penalises a neonatal sample misclassified as adult exactly as much as one misclassified as the adjacent suckling stage—so a developmental stager should additionally respect stage order. Models that regress on the integer-coded stage index (PLS, the ridge family, PCA+ridge) or decompose it into cumulative monotone subtasks (the Frank–Hall ordinal GBM) are order-aware and keep their errors near the diagonal; nominal classifiers—the remaining trees, logistic/SVC/kNN/naive-Bayes/MLP, and PLS-DA, which one-hot encodes the stages—treat the six stages as unordered labels, so nothing prevents a neonatal sample from being sent to a distant stage. **Transfer-capable?** a linear model's score is a weighted sum that can be projected onto a target's 1:1 orthologs (yes for ridge/SVC/elastic-net/PLS/PLS-DA); PCA models (*) require the pig-fit principal-component basis and transfer only partially; trees, kNN and naive Bayes do not. **Biomarker?** PLS and PLS-DA yield the canonical Variable Importance in Projection (VIP); ridge/SVC give weak coefficient weights; trees give split-gain importances that are informative within pig but non-transferable (†); MLP/kNN/NB yield none. **Compute** (peak resident memory and total CPU-time over the run; indicative of order-of-magnitude cost rather than a controlled timing benchmark, since the boosted-tree and SVD-bound models used six BLAS threads while gradient boosting ran single-threaded): the boosted trees that top the honest ranking are also the most expensive (2.3–2.9 GB and many CPU-hours), whereas PLS fits in 1.3 GB and about 11 min. **PLS is the only estimator that is simultaneously order-aware, cross-species transfer-capable and VIP-biomarker-yielding**, and among those three properties it is also the cheapest to run; its 0.11-BA shortfall to the non-transferable tree (0.427 versus 0.537) is modest—both models sit far below their own batch-inclusive scores—and buys neither cross-species transfer nor VIP biomarkers. That the chosen PLS keeps its errors near the diagonal is borne out in practice by its ordinal Spearman correlation on muscle ($\rho = 0.66$ under grouped cross-validation; main text). PLS-regression is preferred over PLS-DA on two counts: PLS-DA one-hot encodes the stages and is therefore not order-aware, and only the continuous ordinal score of PLS-regression is what is projected across species. Both PLS values come from the same fixed 10-component default model (no hyperparameter tuning): this standalone benchmark harness loads muscle as $n = 757$ over 54 BioProjects and reports 0.427, whereas the main-text pipeline (Figs 3, S1; $n = 757$ over 54 BioProjects) reports 0.45 ± 0.04, the two agreeing to within the seed-level scatter. Of twenty estimators attempted, LDA and the soft-vote ensemble did not finish (a ∼27 GB, multi-hour SVD on 31,908 features) and are omitted.

Supplementary Table S7: The pig-trained developmental score is not a re-detected universal proliferation axis: per-cell transfer of pig PLS versus two pig-independent baselines. For every cross-species cell, the pig-trained PLS developmental score (ρ_{PLS}) is compared against two baselines computed on the *identical* per-gene z-scored shared-ortholog target matrix and the identical target ordinal developmental level, but using *no* information from the pig: (i) the target's own unsupervised first principal component ($|\rho_{\mathrm{PC1}}|$; the PC sign is arbitrary, so the absolute correlation is reported), and (ii) a fixed conserved cell-cycle/proliferation index (ρ_{CC}, signed), the equal-weighted mean of the z-scored one-to-one orthologs of the Tirosh *et al.* (2016) S-phase and G2/M signature[66] (96 symbols; n_{CC} present per cell, lower in chicken owing to fewer 1:1 orthologs). ρ_{CC} is negative in nearly every organ because cell-cycle activity declines with maturation, and positive only where germ-cell proliferation rises postnatally (testis). Across all 47 fittable cells the pooled means are close (PLS 0.709, cell-cycle 0.687, PC1 0.670 mean $|\rho|$) and the paired PLS>baseline win-rates are not significant (PLS>PC1 22/47, Wilcoxon $p = 0.86$; PLS>CC 27/47, $p = 0.54$), because in highly proliferative or labile organs (kidney, rodent liver/ovary, testis) a generic target-only index happens to align with the maturation axis. The decisive result is the tissue-identity ranking (Cardoso per-organ mean $|\rho|$): PLS recovers the paper's conserved≫labile ordering (brain 0.92, heart 0.89 ≫ liver 0.49, ovary 0.43), whereas neither baseline reproduces it (rank-Spearman versus PLS: PC1 +0.03, placing heart last; cell-cycle +0.09, placing testis last). This conserved-organ summary row spans the Cardoso + dGTEx Brain+Heart cells (cell-cycle mean 0.73, including the underpowered dGTEx heart cell); the main-text margins (+0.24 versus PC1, +0.14 versus cell-cycle) are computed on the Cardoso-only Brain+Heart cells, whose cell-cycle mean is 0.77. All three methods are divergence-independent (Spearman($|\rho|$, divergence): PLS −0.25, $p = 0.11$; PC1 −0.08; CC −0.00), so the universal-axis hypothesis—which predicts the baselines would track the PLS tissue ordering—is refuted: the baselines re-detect a generic proliferation signal everywhere but fail to recover the tissue-specific conservation gradient that the pig-learned linear programme captures, most clearly in heart and in the well-powered dGTEx lung/muscle/adipose cells.

Substrate	Organ (species)	n_{tgt}	ρ_{PLS}	$\lvert\rho_{\mathrm{PC1}}\rvert$	ρ_{CC}	n_{CC}
Cardoso	Brain (Human)	21	0.86	0.63	−0.57	88
Cardoso	Heart (Human)	10	0.91	0.16	−0.44	88
Cardoso	Kidney (Human)	9	0.63	0.63	−0.82	88
Cardoso	Liver (Human)	12	0.40	0.46	−0.49	88
Cardoso	Testis (Human)	13	0.83	0.85	0.82	88
Cardoso	Brain (Macaque)	18	0.96	0.03	−0.20	85
Cardoso	Heart (Macaque)	21	0.79	0.20	−0.71	85
Cardoso	Kidney (Macaque)	24	0.85	0.19	−0.84	85
Cardoso	Liver (Macaque)	25	0.51	0.07	−0.71	85
Cardoso	Testis (Macaque)	21	0.91	0.85	0.44	85
Cardoso	Brain (Mouse)	20	0.88	0.94	−0.91	90
Cardoso	Heart (Mouse)	20	0.91	0.89	−0.87	90
Cardoso	Kidney (Mouse)	20	0.93	0.89	−0.96	90
Cardoso	Liver (Mouse)	20	0.23	0.94	−0.91	90
Cardoso	Ovary (Mouse)	10	0.81	0.89	−0.94	90
Cardoso	Testis (Mouse)	10	0.91	0.98	−0.74	90
Cardoso	Brain (Rat)	24	0.91	0.96	−0.88	75
Cardoso	Heart (Rat)	23	0.92	0.94	−0.92	75
Cardoso	Kidney (Rat)	24	0.76	0.95	−0.98	75
Cardoso	Liver (Rat)	24	0.67	0.99	−0.93	75
Cardoso	Ovary (Rat)	12	0.88	0.99	−0.81	75
Cardoso	Testis (Rat)	12	0.90	0.95	−0.54	75
Cardoso	Brain (Rabbit)	15	0.95	0.81	−0.79	78
Cardoso	Heart (Rabbit)	14	0.95	0.90	−0.91	78
Cardoso	Kidney (Rabbit)	16	0.91	0.87	−0.89	78
Cardoso	Liver (Rabbit)	15	0.84	0.97	−0.76	78
Cardoso	Ovary (Rabbit)	5	0.32	0.95	−0.95	78
Cardoso	Testis (Rabbit)	6	0.74	0.50	0.12	78
Cardoso	Brain (Opossum)	42	0.90	0.97	−0.97	73
Cardoso	Heart (Opossum)	39	0.83	0.92	−0.77	73
Cardoso	Kidney (Opossum)	24	0.60	0.40	−0.92	73
Cardoso	Liver (Opossum)	43	0.35	0.97	−0.89	73
Cardoso	Ovary (Opossum)	20	0.11	0.47	−0.14	73
Cardoso	Testis (Opossum)	22	0.72	0.85	−0.45	73
Cardoso	Brain (Chicken)	20	0.96	0.88	−0.83	51
Cardoso	Heart (Chicken)	20	0.95	0.12	−0.97	51
Cardoso	Kidney (Chicken)	20	0.74	0.96	−0.93	51
Cardoso	Liver (Chicken)	20	0.45	0.93	−0.83	51
Cardoso	Ovary (Chicken)	10	−0.02	0.05	−0.07	51
Cardoso	Testis (Chicken)	9	0.71	0.64	0.42	51
dGTEx	Lung (Human)	35	0.67	0.26	−0.44	88
dGTEx	Muscle (Human)	48	0.59	0.28	−0.53	88
dGTEx	Adipose (Human)	38	0.66	0.27	−0.41	88
dGTEx	Heart (Human)‡	7	0.77	0.85	−0.15	88
dGTEx	Liver (Human)‡	11	0.33	0.30	−0.47	88
dGTEx	Spleen (Human)‡	8	0.13	0.06	−0.57	88
dGTEx	Testis (Human)‡	10	0.74	0.90	0.67	88
**Mean $\lvert\rho\rvert$, all 47 cells**			0.71	0.67	0.69	
**Mean $\lvert\rho\rvert$, conserved organs (Brain+Heart)**			0.90	0.68	0.73	
**Mean $\lvert\rho\rvert$, labile organs (Kid+Tes+Liv+Ov)**			0.64	0.73	0.69	

‡dGTEx heart/liver/spleen/testis are underpowered ($n_{\mathrm{tgt}} < 12$) and are listed for completeness only; the well-powered dGTEx cells are lung, muscle and adipose. $|\rho_{\mathrm{PC1}}|$ uses the absolute correlation because the principal-component sign is arbitrary; ρ_{CC} is reported signed (its magnitude $|\rho_{\mathrm{CC}}|$ is the quantity entering the means and the win-rate comparisons). All three methods consume the same z-scored shared-ortholog target matrix and target ordinal level, so the comparison is fair; the cell-cycle index and PC1 are pig-independent.

Supplementary Table S8: Conserved marker panel used to test the developmental-stage boundaries. Each marker is a canonical, bulk-detectable developmental gene assayed in the tissue in which it is expressed; the expected direction and pattern class are those predicted by the cited literature, and are tested at the corresponding boundary in Figure 2b. The early boundaries (B1–B3) carry sharp or ramped conserved markers, whereas the pubertal (B4) and adult (B5) boundaries are physiological, literature-defined anchors for which no bulk-mRNA switch is demonstrable on present sampling. This table lists the principal anchor(s) per boundary; Figure 2b,c score the full panel of 14 markers (B1, nine; B2 and B3, one each; B4, two; B5, one), which additionally includes the myofibrillar markers *MYL4* and *TNNT3* [67], the fetal hepatic marker *AFP*, and the Leydig-cell marker *INSL3*.

Boundary	Marker(s)	Tissue	Expected (pattern)	Source
B1 perinatal (∼3 d)	*ALB*	Liver	up (sharp)	[68]
	IGF2, *PEG3*	Liver	down (sharp)	[69]
	MYH3, *MYH8*	Muscle	down (sharp)	[70, 67]
	MYH2	Muscle	up (sharp)	[70]
B2 weaning (∼28 d)	*LCT*	Ileum	down (sharp)	[71]
B3 growth (∼90 d)	*IGF1*	Liver	up (ramp)	[72]
B4 puberty (∼165 d)	*AMH*	Testis	down (ramp)	[73]
B5 adult (∼540 d)	*CDKN2A* (p16$^{\text{INK4a}}$)	Liver	up (weak)	[74]

References

[1] Yazmin I. Rovira Gonzalez et al. "Mss51 deletion enhances muscle metabolism and glucose homeostasis in mice". eng. In: *JCI Insight* 4.20 (Oct. 2019), e122247, 122247. ISSN: 2379-3708. DOI: 10.1172/jci.insight.122247.

[2] Mingshan Xue et al. "Distinct domains of complexin I differentially regulate neurotransmitter release". In: *Nature Structural & Molecular Biology* 14.10 (2007), pp. 949–958. DOI: 10.1038/nsmb1292.

[3] Yasutoshi Ando et al. "Tumor necrosis factor (TNF)-α-induced repression of GKAP42 protein levels through cGMP-dependent kinase (cGK)-Iα causes insulin resistance in 3T3-L1 adipocytes". In: *The Journal of Biological Chemistry* 290.9 (2015), pp. 5881–5892. DOI: 10.1074/jbc.M114.624759.

[4] Junya Kohroki et al. "ASB proteins interact with Cullin5 and Rbx2 to form E3 ubiquitin ligase complexes". In: *FEBS Letters* 579.30 (2005), pp. 6796–6802. DOI: 10.1016/j.febslet.2005.11.016.

[5] L Houghton and N Rosenthal. "Regulation of a muscle-specific transgene by persistent expression of Hox genes in postnatal murine limb muscle". In: *Developmental Dynamics* 216.4–5 (1999), pp. 385–397. DOI: 10.1002/(SICI)1097-0177(199912)216:4/5<385::AID-DVDY7>3.0.CO;2-G.

[6] Beat Trueb. "Biology of FGFRL1, the fifth fibroblast growth factor receptor". In: *Cellular and Molecular Life Sciences* 68.6 (2011), pp. 951–964. DOI: 10.1007/s00018-010-0576-3.

[7] Ruth Amann et al. "The FgfrL1 receptor is required for development of slow muscle fibers". eng. In: *Dev Biol* 394.2 (Oct. 2014), pp. 228–241. ISSN: 1095-564X. DOI: 10.1016/j.ydbio.2014.08.016.

[8] Thomas A Ryan and David A Tumbarello. "Optineurin: A Coordinator of Membrane-Associated Cargo Trafficking and Autophagy". In: *Frontiers in Immunology* 9 (2018), p. 1024. DOI: 10.3389/fimmu.2018.01024.

[9] Jolena N. Waddell et al. "Dlk1 is necessary for proper skeletal muscle development and regeneration". eng. In: *PLoS One* 5.11 (Nov. 2010), e15055. ISSN: 1932-6203. DOI: 10.1371/journal.pone.0015055.

[10] Pushpa Maliekal et al. "Molecular identification of mammalian phosphopentomutase and glucose-1,6-bisphosphate synthase, two members of the alpha-D-phosphohexomutase family". In: *The Journal of Biological Chemistry* 282.44 (2007), pp. 31844–31851. DOI: 10.1074/jbc.M706818200.

[11] Chon-Hwa Tsai-Morris et al. "Gonadotropin-regulated testicular RNA helicase (GRTH/Ddx25) is essential for spermatid development and completion of spermatogenesis". In: *Proceedings of the National Academy of Sciences of the United States of America* 101.17 (2004), pp. 6373–6378. DOI: 10.1073/pnas.0401855101.

[12] Guoqing Xia et al. "Heparan sulfate 3-O-sulfotransferase isoform 5 generates both an antithrombin-binding site and an entry receptor for herpes simplex virus, type 1". In: *The Journal of Biological Chemistry* 277.40 (2002), pp. 37912–37919. DOI: 10.1074/jbc.M204209200.

[13] Yun Liu et al. "Abnormal development of the neuromuscular junction in Nedd4-deficient mice". In: *Developmental Biology* 330.1 (2009), pp. 153–166. DOI: 10.1016/j.ydbio.2009.03.023.

[14] Koji Ikegami et al. "TTLL7 is a mammalian beta-tubulin polyglutamylase required for growth of MAP2-positive neurites". In: *The Journal of Biological Chemistry* 281.41 (2006), pp. 30707–30716. DOI: 10.1074/jbc.M603984200.

[15] Erin F Spence et al. "In vivo proximity proteomics of nascent synapses reveals a novel regulator of cytoskeleton-mediated synaptic maturation". In: *Nature Communications* 10.1 (2019), p. 386. DOI: 10.1038/s41467-019-08288-w.

[16] Pradyut K Paul et al. "Histone chaperone ASF1B promotes human β-cell proliferation via recruitment of histone H3.3". In: *Cell Cycle* 15.23 (2016), pp. 3191–3202. DOI: 10.1080/15384101.2016.1241914.

[17] D J Baker et al. "Mitotic regulation of the anaphase-promoting complex". In: *Cellular and Molecular Life Sciences* 64.5 (2007), pp. 589–600. DOI: 10.1007/s00018-007-6443-1.

[18] Leticia Fröhlich Archangelo et al. "The CATS (FAM64A) protein is a substrate of the Kinase Interacting Stathmin (KIS)". In: *Biochimica et Biophysica Acta (BBA) - Molecular Cell Research* 1833.5 (2013), pp. 1269–1279. DOI: 10.1016/j.bbamcr.2013.02.004.

[19] Nagi G. Ayad et al. "Tome-1, a trigger of mitotic entry, is degraded during G1 via the APC". eng. In: *Cell* 113.1 (2003), pp. 101–113. DOI: 10.1016/s0092-8674(03)00232-0.

[20] Vibhu Sahni et al. "Crim1 and Kelch-like 14 exert complementary dual-directional developmental control over segmentally specific corticospinal axon projection targeting". In: *Cell Reports* 37.3 (2021), p. 109842. DOI: 10.1016/j.celrep.2021.109842.

[21] Rebecca Greenlees et al. "Mutations in SIPA1L3 cause eye defects through disruption of cell polarity and cytoskeleton organization". In: *Human Molecular Genetics* 24.20 (2015), pp. 5789–5804. DOI: 10.1093/hmg/ddv298.

[22] Tatsuya Takemoto et al. "Tbx6-dependent Sox2 regulation determines neural or mesodermal fate in axial stem cells". In: *Nature* 470.7334 (2011), pp. 394–398. DOI: 10.1038/nature09729.

[23] T. John Martin. "PTH1R Actions on Bone Using the cAMP/Protein Kinase A Pathway". In: *Frontiers in Endocrinology* 12 (2022), p. 833221. DOI: 10.3389/fendo.2021.833221.

[24] Henry M. Kronenberg. "Developmental regulation of the growth plate". In: *Nature* 423.6937 (2003), pp. 332–336. DOI: 10.1038/nature01657.

[25] Adam Williamson et al. "Identification of a physiological E2 module for the human anaphase-promoting complex". In: *Proceedings of the National Academy of Sciences* 106.43 (2009), pp. 18213–18218. DOI: 10.1073/pnas.0907887106.

[26] Chanlu Xie et al. "Ubiquitin-conjugating enzyme E2C: a potential cancer biomarker". In: *The International Journal of Biochemistry & Cell Biology* 47 (2014), pp. 113–117. DOI: 10.1016/j.biocel.2013.11.023.

[27] Anja Hagting et al. "Human securin proteolysis is controlled by the spindle checkpoint and reveals when the APC/C switches from activation by Cdc20 to Cdh1". In: *The Journal of Cell Biology* 157.7 (2002), pp. 1125–1137. DOI: 10.1083/jcb.200111001.

[28] Esther E. Creemers et al. "Coactivation of MEF2 by the SAP domain proteins myocardin and MASTR". In: *Molecular Cell* 23.1 (2006), pp. 83–96. DOI: 10.1016/j.molcel.2006.05.026.

[29] Stryder M. Meadows et al. "The myocardin-related transcription factor, MASTR, cooperates with MyoD to activate skeletal muscle gene expression". In: *Proceedings of the National Academy of Sciences* 105.5 (Feb. 2008), pp. 1545–1550. DOI: 10.1073/pnas.0703918105.

[30] M. H. Schwab et al. "Neuronal Basic Helix-Loop-Helix Proteins (NEX, neuroD, NDRF): Spatiotemporal Expression and Targeted Disruption of the NEX Gene in Transgenic Mice". In: *The Journal of Neuroscience* 18.4 (1998), pp. 1408–1418. DOI: 10.1523/JNEUROSCI.18-04-01408.1998.

[31] Shuo Yang et al. "Tastin is required for bipolar spindle assembly and centrosome integrity during mitosis". In: *The FASEB Journal* 22.6 (2008), pp. 1960–1972. DOI: 10.1096/fj.07-081463.

[32] Sergei S. Nikonov et al. "Cones Respond to Light in the Absence of Transducin β Subunit". In: *The Journal of Neuroscience* 33.12 (2013), pp. 5182–5194. DOI: 10.1523/JNEUROSCI.5204-12.2013.

[33] Stefania Artigiani et al. "Plexin-B3 is a functional receptor for semaphorin 5A". In: *EMBO Reports* 5.7 (2004), pp. 710–714. DOI: 10.1038/sj.embor.7400189.

[34] Takahiko Hara and Yuki Nakayama. "CXCL14 and insulin action". In: *Vitamins and Hormones* 80 (2009), pp. 107–123. DOI: 10.1016/S0083-6729(08)00605-5.

[35] Wenxia Jiang et al. "Mapping the functional domains of TCblR/CD320, the receptor for cellular uptake of transcobalamin-bound cobalamin". In: *FASEB Journal* 27.8 (2013), pp. 2988–2994. DOI: 10.1096/fj.13-230185.

[36] Clara Marco-Marín et al. "Δ1 -Pyrroline-5-carboxylate synthetase deficiency: An emergent multi-faceted urea cycle-related disorder". eng. In: *J Inherit Metab Dis* 43.4 (July 2020), pp. 657–670. ISSN: 1573-2665. DOI: 10.1002/jimd.12220.

[37] R. Benezra et al. "The protein Id: a negative regulator of helix-loop-helix DNA binding proteins". In: *Cell* 61.1 (Apr. 1990), pp. 49–59. ISSN: 0092-8674. DOI: 10.1016/0092-8674(90)90214-y.

[38] Jonathan Perk, Antonio Iavarone, and Robert Benezra. "Id family of helix-loop-helix proteins in cancer". In: *Nature Reviews Cancer* 5.8 (2005), pp. 603–614. DOI: 10.1038/nrc1673.

[39] Rong-Zong Liu et al. "CRABP1 is associated with a poor prognosis in breast cancer: adding to the complexity of breast cancer cell response to retinoic acid". In: *Molecular Cancer* 14 (2015), p. 129. DOI: 10.1186/s12943-015-0380-7.

[40] Z. Nikolic et al. "The human glycine receptor subunit alpha3. Glra3 gene structure, chromosomal localization, and functional characterization of alternative transcripts". In: *J Biol Chem* 273.31 (July 1998), pp. 19708–19714. ISSN: 0021-9258. DOI: 10.1074/jbc.273.31.19708.

[41] Jos P. M. van Putten and Karin Strijbis. "Transmembrane Mucins: Signaling Receptors at the Intersection of Inflammation and Cancer". In: *Journal of Innate Immunity* 9.3 (2017), pp. 281–299. DOI: 10.1159/000453594.

[42] Tuanlao Wang and Wanjin Hong. "Interorganellar regulation of lysosome positioning by the Golgi apparatus through Rab34 interaction with Rab-interacting lysosomal protein". In: *Molecular Biology of the Cell* 13.12 (2002), pp. 4317–4332. DOI: 10.1091/mbc.e02-05-0280.

[43] Yingbao Wang et al. "Novel Adipokine, FAM19A5, Inhibits Neointima Formation After Injury Through Sphingosine-1-Phosphate Receptor 2". In: *Circulation* 138.1 (2018), pp. 48–63. DOI: 10.1161/CIRCULATIONAHA.117.032398.

[44] Peter M. H. Heegaard et al. "Pig alpha1-acid glycoprotein: characterization and first description in any species as a negative acute phase protein". In: *PLoS ONE* 8.7 (2013), e68110. DOI: 10.1371/journal.pone.0068110.

[45] S. Perrin and J. A. Foster. “Developmental regulation of elastin gene expression”. In: *Critical Reviews in Eukaryotic Gene Expression* 7.1-2 (1997), pp. 1–10. DOI: 10.1615/critreveukargeneexpr.v7.i1-2.10.

[46] Björn Meyer et al. “Identification of two proteins associated with mammalian ATP synthase”. In: *Molecular & Cellular Proteomics* 6.10 (2007), pp. 1690–1699. DOI: 10.1074/mcp.M700097-MCP200.

[47] Y Wang et al. “Rim is a putative Rab3 effector in regulating synaptic-vesicle fusion”. eng. In: *Nature* 388.6642 (Aug. 1997), pp. 593–598. DOI: 10.1038/41580.

[48] Tim Ziera et al. “Cnksr3 is a direct mineralocorticoid receptor target gene and plays a key role in the regulation of the epithelial sodium channel”. In: *FASEB Journal* 23.11 (2009), pp. 3936–3946. DOI: 10.1096/fj.09-134759.

[49] Nadège Vernet et al. “Mouse Y-linked Zfy1 and Zfy2 are expressed during the male-specific interphase between meiosis I and meiosis II and promote the 2nd meiotic division”. In: *PLoS Genetics* 10.6 (2014), e1004444. DOI: 10.1371/journal.pgen.1004444.

[50] C Mertens et al. “Desmosomal plakophilin 2 as a differentiation marker in normal and malignant tissues”. In: *Differentiation* 64.5 (1999), pp. 277–290. DOI: 10.1046/j.1432-0436.1999.6450277.x.

[51] Alexander K Godfrey et al. “Quantitative analysis of Y-Chromosome gene expression across 36 human tissues”. In: *Genome Research* 30.6 (2020), pp. 860–873. DOI: 10.1101/gr.261248.120.

[52] Thomas Rival et al. “Inner-membrane proteins PMI/TMEM11 regulate mitochondrial morphogenesis independently of the DRP1/MFN fission/fusion pathways”. eng. In: *EMBO Reports* 12.3 (2011), pp. 223–230. DOI: 10.1038/embor.2010.214.

[53] Rafael K. Campos et al. “RPLP1 and RPLP2 Are Essential Flavivirus Host Factors That Promote Early Viral Protein Accumulation”. In: *Journal of Virology* 91.4 (2017), e01706–16. DOI: 10.1128/JVI.01706-16.

[54] Ruijuan Xu et al. “Golgi alkaline ceramidase regulates cell proliferation and survival by controlling levels of sphingosine and S1P”. In: *FASEB Journal* 20.11 (2006), pp. 1813–1825. DOI: 10.1096/fj.05-5689com.

[55] Helena Hauge, Sebastian Patzke, and Hans-Christian Aasheim. “Characterization of the FAM110 gene family”. eng. In: *Genomics* 90.1 (2007), pp. 14–27. DOI: 10.1016/j.ygeno.2007.03.002.

[56] Anastasia Georgountzou and Nikolaos G. Papadopoulos. “Postnatal Innate Immune Development: From Birth to Adulthood”. In: *Front Immunol* 8 (Aug. 2017), p. 957. ISSN: 1664-3224. DOI: 10.3389/fimmu.2017.00957.

[57] F. E. Johansen, R. Braathen, and P. Brandtzaeg. "Role of J chain in secretory immunoglobulin formation". In: *Scandinavian Journal of Immunology* 52.3 (Sept. 2000), pp. 240–248. ISSN: 0300-9475. DOI: 10.1046/j.1365-3083.2000.00790.x.

[58] G C Starling et al. "Characterization of mouse CD6 with novel monoclonal antibodies which enhance the allogeneic mixed leukocyte reaction". In: *European Journal of Immunology* 26.4 (1996), pp. 738–746. DOI: 10.1002/eji.1830260403.

[59] G. F. Gao and B. K. Jakobsen. "Molecular interactions of coreceptor CD8 and MHC class I: the molecular basis for functional coordination with the T-cell receptor". In: *Immunology Today* 21.12 (2000), pp. 630–636. DOI: 10.1016/s0167-5699(00)01750-3.

[60] C. L. Sommers et al. "Function of CD3 epsilon-mediated signals in T cell development". In: *The Journal of Experimental Medicine* 192.6 (2000), pp. 913–919. DOI: 10.1084/jem.192.6.913.

[61] Glen M. Corson et al. "Differential expression of fibrillin-3 adds to microfibril variety in human and avian, but not rodent, connective tissues". In: *Genomics* 83.3 (2004), pp. 461–472. DOI: 10.1016/j.ygeno.2003.08.023.

[62] Huilin Huang et al. "Recognition of RNA N6-methyladenosine by IGF2BP proteins enhances mRNA stability and translation". In: *Nature Cell Biology* 20.3 (2018), pp. 285–295. DOI: 10.1038/s41556-018-0045-z.

[63] Najla Fiaturi et al. "CCN5 in alveolar epithelial proliferation and differentiation during neonatal lung oxygen injury". In: *Journal of Cell Communication and Signaling* 12.1 (2018), pp. 217–229. DOI: 10.1007/s12079-017-0443-1.

[64] Luca Simeoni et al. "The transmembrane adapter protein SIT regulates thymic development and peripheral T-cell functions". In: *Molecular and Cellular Biology* 25.17 (2005), pp. 7557–7568. DOI: 10.1128/MCB.25.17.7557-7568.2005.

[65] Andrés Alcover, Balbino Alarcón, and Vincenzo Di Bartolo. "Cell Biology of T Cell Receptor Expression and Regulation". In: *Annual Review of Immunology* 36 (2018), pp. 103–125. DOI: 10.1146/annurev-immunol-042617-053429.

[66] Itay Tirosh et al. "Dissecting the multicellular ecosystem of metastatic melanoma by single-cell RNA-seq". eng. In: *Science* 352.6282 (Apr. 2016), pp. 189–196. ISSN: 1095-9203. DOI: 10.1126/science.aad0501.

[67] Stefano Schiaffino and Carlo Reggiani. "Fiber Types in Mammalian Skeletal Muscles". In: *Physiological Reviews* 91.4 (2011), pp. 1447–1531. DOI: 10.1152/physrev.00031.2010.

[68] B. T. Spear et al. “Transcriptional control in the mammalian liver: liver development, perinatal repression, and zonal gene regulation”. In: *Cellular and Molecular Life Sciences* 63.24 (2006), pp. 2922–2938. DOI: 10.1007/s00018-006-6258-5.

[69] Julian C. Lui et al. “An imprinted gene network that controls mammalian somatic growth is down-regulated during postnatal growth deceleration in multiple organs”. In: *American Journal of Physiology-Regulatory, Integrative and Comparative Physiology* 295.1 (2008), R189–R196. DOI: 10.1152/ajpregu.00182.2008.

[70] G Fazarinc et al. “Dynamics of myosin heavy chain isoform transition in the longissimus muscle of domestic and wild pigs during growth: a comparative study”. In: *Animal : an international journal of animal bioscience* 11.1 (2017), pp. 164–174. DOI: 10.1017/S1751731116001312.

[71] D. J. Hampson and D. E. Kidder. “Influence of creep feeding and weaning on brush border enzyme activities in the piglet small intestine”. In: *Research in Veterinary Science* 40.1 (1986), pp. 24–31.

[72] D G Burrin et al. “Role of milk-borne vs endogenous insulin-like growth factor I in neonatal growth.” In: *Journal of Animal Science* 75.10 (1997), pp. 2739–2743. DOI: 10.2527/1997.75102739x.

[73] A. Sansone et al. “AMH and INSL3 in testicular and extragonadal pathophysiology: what do we know?” In: *Andrology* 7.2 (2019), pp. 131–138. DOI: 10.1111/andr.12597.

[74] J Krishnamurthy et al. “Ink4a/Arf expression is a biomarker of aging”. In: *The Journal of clinical investigation* 114.9 (2004), pp. 1299–1307. DOI: 10.1172/JCI22475.
